# Biomarker Candidates for Tumors Identified from Deep-Profiled Plasma Stem Predominantly from the Low Abundant Area

**DOI:** 10.1021/acs.jproteome.2c00122

**Published:** 2022-05-23

**Authors:** Marco Tognetti, Kamil Sklodowski, Sebastian Müller, Dominique Kamber, Jan Muntel, Roland Bruderer, Lukas Reiter

**Affiliations:** Biognosys, Schlieren, Zurich 8952, Switzerland

**Keywords:** plasma proteomics, data-independent acquisition, SWATH, label-free quantification, stable isotope-based quantification, library, single shot, high throughput, clinical proteomics, cancer, depletion

## Abstract

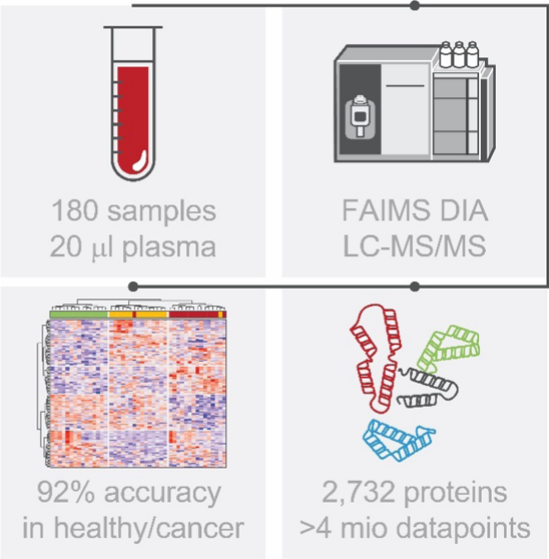

The plasma proteome has the potential to enable a holistic analysis of the health state of an individual. However, plasma biomarker discovery is difficult due to its high dynamic range and variability. Here, we present a novel automated analytical approach for deep plasma profiling and applied it to a 180-sample cohort of human plasma from lung, breast, colorectal, pancreatic, and prostate cancers. Using a controlled quantitative experiment, we demonstrate a 257% increase in protein identification and a 263% increase in significantly differentially abundant proteins over neat plasma. In the cohort, we identified 2732 proteins. Using machine learning, we discovered biomarker candidates such as STAT3 in colorectal cancer and developed models that classify the diseased state. For pancreatic cancer, a separation by stage was achieved. Importantly, biomarker candidates came predominantly from the low abundance region, demonstrating the necessity to deeply profile because they would have been missed by shallow profiling.

## Introduction

Proteins control most biological processes in life. Alterations in their expression level, localization, and proteoforms are often correlated with disease onset and progression.^1^ In humans and animals, blood flows through virtually all tissues. Therefore, it has the potential to indicate the health state of any inner organ, even those not accessible from the outside. Blood is readily obtainable with minimal invasive sampling, and large biobanks exist for retrospective analyses.^2^ Clinical analysis of blood is the most widespread diagnostic procedure in medicine, and blood biomarkers are used to diagnose diseases, categorize patients, and support treatment decisions. While proteins (6–8%) are by far the second major component of plasma after water (90–92%), metabolic, lipidomic, transcriptomic, and genomic readouts are also gaining traction as diagnostic tests in plasma.^3−6^ Different omics readouts can be used in conjunction to improve diagnostic power.^7^ Despite more than 20,000 diseases reported to affect humans,^8^ it is only for a small fraction of them that accurate, sensitive, and specific diagnostic tests exist.

The limited success of blood protein biomarkers is primarily due to analytical challenges that come with the proteomic analysis of blood plasma. On the one hand, the large biological variance between individuals and within individuals over time makes the discovery of reliable biomarker signatures difficult.^9−12^ Further, the steep dynamic range of human plasma, with an estimated dynamic range of 12–13 orders of magnitude,^13^ renders comprehensive proteome profiling challenging to any analytical technique. In the lower concentration range, thousands of proteins reside, mostly tissue leakage proteins and signaling molecules that could serve as biomarkers but are very challenging to measure, especially in an unbiased manner.^14,15^

Mass spectrometry (MS)-based plasma analysis provides an unbiased, quantitative, and therefore ideal technology for the system-wide characterization of the proteome.^16^ Recently, technological developments in sample preparation, chromatography, and acquisition enabled automated, large-scale plasma projects of hundreds of specimens that have resulted in reproducible findings.^15,17−20^ These approaches share the shallow depth of proteome coverage, reaching a maximum of about 600 proteins identified and quantified in a sample. From qualitative analysis, disproportionately more proteins were found to be present in the lower abundance region of plasma than in the higher concentration range.^14^ Novel MS-based approaches have been developed to improve analytical depth while retaining quantitative information. These include the depletion of high-abundance proteins, the enrichment of low abundant proteins of interest, and prefractionation.^21^ Still, they have yet to reach the throughput level needed to measure larger cohorts of clinical samples. Automatization and depletion, batch, and quality control have been tackeled^18,22,23^ but require further improvement for large-scale studies. In summary, while current plasma proteome biomarker research approaches mostly cover the first few hundred proteins by concentration, rigorous experimental design and comprehensive, large-scale quantitative studies will achieve generalizable biomarker discovery.^16^

Screening for the most common cancer types cannot be done in a routine and population-wide manner. To date, only a few nonideal, validated biomarkers exist in clinical use.^24^ A significant challenge is that generally, only a single analyte or metric is measured despite the known heterogeneity of cancer. Biomarkers that accurately enable early detection in asymptotic subjects, reflect cancer aggressiveness at diagnosis, and improve risk stratification are urgently needed.^24^ Despite the medical need, plasma biomarker candidates for cancer are rarely validated or transferred to the clinic. Recent examples are as follows: Zhang et al. performed discovery proteomics in the plasma of 10 patients with colorectal cancer, discovered 72 biomarker candidates, and then performed a successful follow-up verification for prognostic markers with 419 patients using an immunoassay.^25,26^ Enroth et al. found plasma protein biomarker signatures for ovarian cancer^27^ but performed no validation. He et al. showed that for hepatocellular carcinoma and cholangiocarcinoma, biomarker candidates could be identified from plasma; the validation of these candidates is still pending.^28^ Zhou et al. identified biomarkers for early gastric cancer from a small sample set, but validation is still pending.^29^ For prostate cancer, a blood diagnostic test was successfully developed based on the discovery of proteomics and is now being used in the clinic.^30^ For the detection of early ovarian cancer, the OVA1 test was developed and approved, where the measurement of β-2 macroglobulin, apolipoprotein 1, serum transferrin, and prealbumin is combined with the previously established marker CA125 to deliver better care.^31,32^ This case exemplifies that multimeasurement techniques are expected to outperform single biomarker panels. Furthermore, single protein biomarkers are rarely specific for a single disease, e.g., α fetoprotein is diagnostic in liver cancer, but the biomarker is not specific, as it is altered in other liver diseases and ovarian and testis cancers.^33^ Rarely, there are highly specific biomarkers such as β subunit HCG (β-HCG), which is a serum marker for testicular carcinoma as β-HCG is never detected in the circulation of healthy males.^34^ To make plasma biomarker discovery more efficient and successful, the comprehensive profiling and validation of large cohorts of plasma proteomes need to be significantly improved with new approaches.^16^ The expected outcome is new biomarkers that will allow early cancer detection and prediction of the probable response to therapy (in precision medicine).

We demonstrate a novel, automated analytical approach for plasma profiling to a depth of 2732 proteins in the presented cancer study and identifying deep into tissue leakage and signaling molecular areas. We demonstrate the identification and quantitative benefits of neat plasma profiling through a controlled quantitative experiment. Further, we profiled deep into the tissue leakage plasma samples coming from both healthy patients and patients with one of the five most deadly solid tumors in the United States.^35^ A biomarker analysis with machine learning revealed candidates and models able to classify healthy and diseased samples. The discovered biomarker candidates predominantly came from low abundance protein regions, clearly demonstrating the need to measure deeply because they would have been missed by shallow plasma profiling.

## Experimental Procedures

### Ethics

The Cantonal Ethics Committee for Research on Human Beings, Zürich, Switzerland, approved the study protocol to be performed (proteomic analysis of plasma samples (2020-02892)).

### Cohort Selection and Study Design

Cohort selection and experimental design were driven by sample availability in commercial repositories. For each cancer type, 30 matching samples were selected and split into early (nonmetastatic stages IA–IIC) and late (nonmetastatic stage IIIA–C) groups. Prior to the analysis, normal individuals were matched for age, sex, and whenever possible balanced across ethnicities to both early and late groups for each cancer type. Healthy samples are self-declared healthy. This resulted in three equal control groups (*n* = 15) with overlapping individuals, namely, breast cancer control, prostate cancer control, and remaining cancer control. Matching was done manually using the χ2 test or ANOVA with a *p*-value threshold of 0.05 (R-package “tableone”).

### Sample Preparation of the Pan-Cancer Cohort

One hundred and eighty human plasma samples were obtained from Precision for Medicine and its subsidiaries (Norton USA), Discovery Life Sciences (Huntsville), and ProteoGenex (Los Angeles). Due to limited availability, samples were not balanced across suppliers; collection procedures and handling until storage at −80 °C are considered to be the same in the case of all three providers (Supporting Information Table 1). All samples were handled equally and thawed twice. During the aliquoting, a small amount of each sample was pooled. This quality control sample was subsequently used for the library generation and to assess the quality and batch effects throughout the sample preparation and acquisition. The processing batches were block-randomized for disease status, diseased state, gender, and ethnicity (only relevant for breast cancer samples) and kept for the entire sample preparation.

Depletion was performed using the Agilent multi affinity removal column human-14, 4.6 × 50 mm^2^ (Agilent Technologies) set up on a Dionex Ultimate 3000 RS pump (Thermo Fisher Scientific) and run according to the manufacturer’s instructions. Briefly, the plasma was diluted 4:1 with buffer A for multiple affinity removal LC columns (Agilent Technologies) and filtered through a 0.22 μm hydrophilic PVDF membrane filter plate (Millipore) before 70 μL was injected onto the column. The gradient was 27.5 min long, with the collection occurring between 3.6 and 9.2 min, a flow rate of 1 mL/min during 11 and 26.5 min and 0.125 mL/min during the rest of the gradient, and buffer B for multiple affinity removal LC columns (Agilent Technologies) only in the time period of 13–17.5 min (100% buffer B). Well-spaced within each processing batch, we depleted the quality control sample three times and treated it as a separate sample thereon (depletion control samples).

Following depletion, we digested the samples with protein aggregation capture using a KingFisher Flex (Thermo Fisher Scientific).^36^ To assess digestion reproducibility, we mixed two extra depletions of the quality control sample before splitting it into digestion triplicate (digestion control samples). The acidified peptide mixtures were loaded for clean-up into MacroSpin C18 96-well plates (The Nest Group), desalted, and eluted with 50% acetonitrile. Samples were dried in a vacuum centrifuge and solubilized in 0.1% formic acid and 1% acetonitrile with Biognosys’s iRT and PQ500 kits (Biognosys) spiked following the manufacturer’s instruction. Prior to DIA mass spectrometric analyses, the sample’s peptide concentrations were determined using a UV/vis spectrometer at 280 nm/430 nm (SPECTROstar Nano, BMG Labtech) and centrifuged at 14,000*g* at 4 °C for 30 min.

### Sample Preparation of the Controlled Quantitative Experiment

The controlled quantitative experiment was generated from 20 healthy human EDTA K3 plasma samples obtained from Sera Laboratories International Ltd. (West Sussex, U.K.). *Saccharomyces cerevisiae* (*S. cerevisiae*) was lysed in 100 mM HEPES pH 7.4, 150 mM KCl, 1 mM MgCl_2_, by shear force passing through a gauge 12 syringe 15 times on ice before filtering (0.2 μm). *Escherichia coli* (*E. coli*) was lysed with a cell cracker before filtering (0.2 μm). After protein concentration determination using a UV/vis spectrometer at 280 nm (SPECTROstar Nano, BMG Labtech), each sample was spiked with fixed ratios of *E. coli* and *S. cerevisiae* leading to a synthetic 1:2- and 4:3-fold change, respectively. To 20 μL of plasma (∼1200 μg proteins), 40 or 30 μg of *S. cerevisiae* and 12 or 24 μg of *E. coli* lysate were added for conditions A and B, respectively. The resulting 40 samples were diluted 4:1 with buffer A for multiple affinity removal LC columns (Agilent Technologies), filtered through a 0.22 μm hydrophilic PVDF membrane filter plate (Millipore). Seventy microliters was used for depletion as described above followed by filter-aided sample preparation (FASP)^37^ and 30 μL for the neat plasma comparison. The diluted neat plasma sample was precipitated by adding four excesses of cold acetone (v/v) and overnight incubation at −20 °C. The pellet was subsequently washed twice with cold 80% acetone in water (v/v). After air-drying the pellet, the proteins were resuspended in 50 μL denaturation buffer (8 M urea, 20 mM TCEP, 40 mM CAA, 0.1 M ABC), sonicated for 5 min (Bioruptor Plus, Diagenode, 5 cycles high, 30 s on, 30 s off), and incubated at 37 °C for 60 min. Upon dilution with 0.1 M ABC to a final urea concentration of 1.4 M, the samples were digested overnight with a 2 μg sequencing-grade trypsin (Promega) and trypsin inactivated by adding TFA to a final concentration of 1% v/v. Peptide clean-up was carried out as described above.

### Library Generation

High pH reverse-phase (HPRP) fractionation was performed using a Dionex UltiMate 3,000 RS pump (Thermo Fisher Scientific) on an Acquity UPLC CSH C18 1.7 μm, 2.1 × 150 mm^2^ column (Waters) at 60 °C with a 0.3 mL/min flow rate. Prior to loading, the pH of 300 μg of pooled depleted samples was adjusted to pH 10 by adding ammonium hydroxide. The used gradient was 1–40% solvent B in 30 min; solvents were A: 20 mM ammonium formate in water, B: acetonitrile. Fractions were taken every 30 s and sequentially pooled to 20 fraction pools. The fraction pools were then dried down and resuspended in 0.1% formic acid and 1% acetonitrile with Biognosys’s iRT kits spiked according to the manufacturer’s instruction. Before data-dependent acquisition (DDA) mass spectrometric analyses, peptide concentrations were determined, and the samples were centrifuged as described above.

### Mass Spectrometric Acquisition

For data-independent acquisition (DIA) LC-MS measurements for the controlled quantitative experiment, 1 μg of peptides per sample was injected onto an in-house-packed reverse-phase column (PicoFrit emitter) with a 75 μm inner diameter, 60 cm length, and 10 μm tip from New Objective, packed with the Reprosil Saphir C18 1.5 μm phase (Dr. Maisch, Ammerbuch, Germany) on a Thermo Fisher Scientific EASY-nLC 1,200 nanoliquid chromatography system connected to a Thermo Fisher Scientific Orbitrap Exploris 480 mass spectrometer equipped with a Nanospray Flex ion source. The DIA method was adopted from Bruderer et al.^38^ and consisted of one full-range MS1 scan and 29 DIA segments.

For DDA and DIA LC-FAIMS-MS/MS measurements, 4 μg of each sample was separated using a self-packed analytical PicoFrit column (75 μm × 50 cm length) (New Objective, Woburn, MA) packed with ReproSil Saphir C18 1.5 μm (Dr. Maisch GmbH, Ammerbuch, Germany) with a 2 h segmented gradient using an EASY-nLC 1200 (Thermo Fisher Scientific). LC solvents were A: water with 0.1% FA; B: 20% water in acetonitrile with 0.1% FA. For the 2 h gradient, a nonlinear LC gradient was 1–59% solvent B in 120 min followed by 59–90% B in 10 s, 90% B for 8 min, 90 to 1% B in 10 s and 1% B for 5 min at 60 °C, and a flow rate of 250 nL/min. The samples were acquired on an Orbitrap Exploris 480 mass spectrometer (Thermo Fisher Scientific) equipped with a FAIMS Pro device (Thermo Fisher Scientific) using methods based on ref (39). If not specified differently, the FAIMS-DIA method contained three FAIMS CV (−35, −55, and −75 V) parts, each with a survey scan of 120,000 resolution with 20 ms max IT and an AGC of 3 × 10^6^ and 35 DIA segments of 15,000 resolution with IT set to auto and AGC set to custom 1000%. The mass range was set to 350–1650*m*/*z*, the default charge state was set to 3, the loop count was set to 1, and the normalized collision energy was set to 30. For the acquisition of the fractionated sample for the library, a DDA method was applied. The DDA method consisted of three FAIMS CVs (−35, −55, and −75 V): each contained a DDA experiment with 60,000 resolution of MS1, 15,000 resolution of MS2, with a fixed cycle time (1.3 s), IT set to AUTO, and AGC set to custom 500%.^40^

### Mass Spectrometric Data Analysis

#### Database Search for Library Generation

DIA and DDA mass spectrometric data were analyzed using software SpectroMine (version 3.0.2101115.47784, Biognosys) using the default settings, including a 1% false discovery rate control at PSM, peptide, and protein levels, allowing for two missed cleavages and variable modifications (N-term acetylation and methionine oxidation). The human UniProt.fasta database (*Homo sapiens*, 2020-07-01, 20,368 entries) was used, and for the library generation, the default settings were used except for the use of a top 300 precursors per protein filter.

#### Quantitative Analysis of Data-Independent Acquisition

Raw mass spectrometric data were first converted using the HTRMS Converter (version 14.3.200701.47784, Biognosys) and then analyzed using software Spectronaut (version 15.0.210108, Biognosys) with the default settings, but Q-value sparse filtering was enabled with a global imputing strategy and a hybrid library comprising all DIA and DDA runs conducted in this study.^41^ The imputing strategy defines how to estimate the missing values (identifications not fulfilling the FDR threshold), and with the global imputing strategy, the missing values are imputed based on random sampling from a distribution of low abundant signals taken across the entire experiment (lowest 10th percentile ±1 standard deviation).^42^ Default settings include peptide and protein level false discovery rate control at 1% and cross-run normalization using global normalization on the median. Including a high number of quality control samples (depletion, digestion, and injection controls) enabled the investigation of batch effects and quantification of the introduced variability at each step. No batch effect was identified by either principal component analysis (PCA, “stats” R-package) or hierarchical clustering.

CQE DIA data were analyzed using the directDIA approach of Spectronaut software (version 15.0.210108, Biognosys) using the default settings, including a 1% false discovery rate control at PSM, peptide, and protein levels, allowing for two missed cleavages and variable modifications (N-term acetylation and methionine oxidation). The directDIA approach within Spectronaut is an implementation with minor improvements of the published DIA Umpire approach.^43^ The combined human, *E. coli*, and *S. cerevisiae*.fasta databases with the removal of the overlapping tryptic sequences (*Homo sapiens* 2020-08-31, 96,996 entries; *Saccharomyces cerevisiae* (strain ATCC 204508/S288c), 6078 entries; *Escherichia coli* (strain K12), 4857 entries; *Combined*, 96,637 entries) were used, and for the library generation, the default settings were used except for the Q-value sparse filtering enabled with a global imputing strategy and cross-run normalization using global normalization on the median based solely on the human identifications.

When we use proteins, we refer to protein groups as determined by the ID picker algorithm^44^ and implemented in Spectronaut.

#### Data Analysis and Biomarker Selection

Initial univariate candidate filtering was performed using the pairwise Wilcoxon test applied per protein across disease status (healthy, early, and late stages) with the Holmes–Bonferroni correction (within-group). Proteins with a p-value below or equal to 0.05 from the randomly selected 80% of observations were used for further optimization using sparse partial least-squares discriminant analysis (sPLSDA).^45^ A leave-one-out algorithm was used for optimal component and protein selection. sPLSDA training and testing were performed using the R-package “mixOmics”.^46^ The remaining 20% of observations were used for validation. The accuracy of prediction for all three groups, healthy, early, late stages, and healthy against early and late stages together, was calculated as the ratio of the true positive and negative sum of all observations (R-package “caret”). Unsupervised hierarchical analysis was performed with Manhattan distance and Ward’s clustering on centered and normalized data (xij-x̅j/sj, i-th observation with j-th protein) using R-package “ComplexHeatmap”. PCA analysis was performed using R-package “stats”. Correlation analysis was performed using the Pearson correlation with R-packages “stats” and “corrplot”. Correlation significance was tested using a two-sided t-test at 0.05 α. All analyses were performed using log_2_-transformed data. Gene ontology enrichment was performed using GOrilla,^47^ and the identifications of this study were selected as the background. All basic calculations and data transformations were performed in R with R-packages: “dplyr” and “ggplot2”.

## Results

### Optimization and Validation of the Analytical Approach

**Figure 1:**
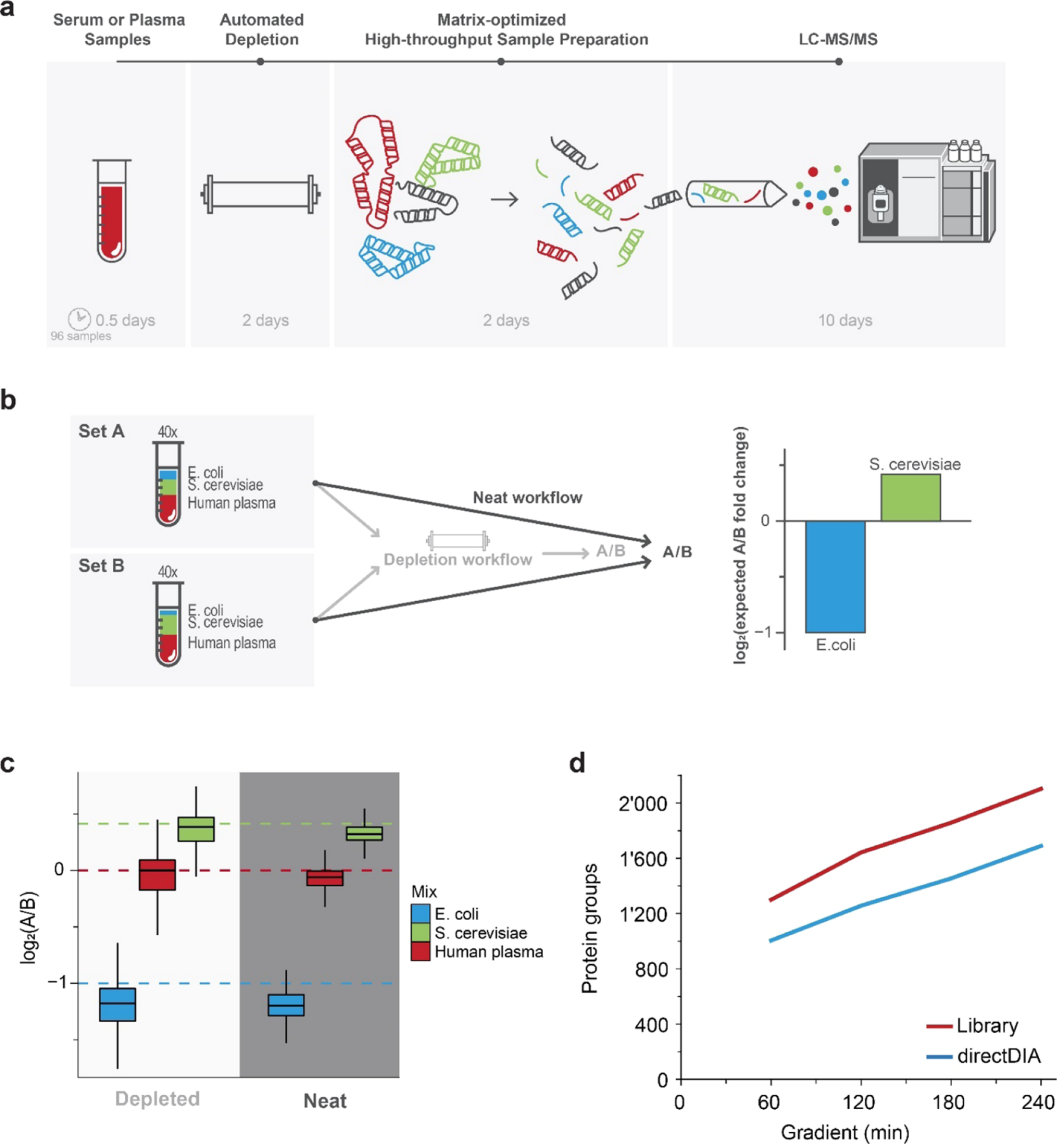
Deep plasma profiling: automated analytical approach and benchmarking. (a) Sketch of the major steps of the analytical approach developed for deep human plasma profiling for biomarker discovery, including the depletion of the 14 most abundant proteins and the approximate time requirements. (b) Schema of the controlled quantitative experiment based on human plasma spiked with known amounts of *Saccharomyces cerevisiae* (*S. cerevisiae*) (1:1.3) and *Escherichia coli* (*E. coli*). (1:.5). The controlled mixtures were either directly digested or processed using the process described in panel (a). (c) Plot showing the measured distributions of the fold changes of the controlled quantitative experiment divided by species. The dashed lines represent the theoretical fold change. (d) Comparison of the number of protein groups identified at different gradient lengths for a depleted human plasma pool by either directDIA (blue) or with a sample-specific library (red).

While methods to analyze the plasma proteome in-depth exist, they are usually either targeted and therefore biased, as for the case of antibody- or aptamer-based technologies, or are based on the principle of fractionation and are therefore difficult to scale. We aimed to develop an analytical method that provided deep coverage and quantitative accuracy while minimizing sample handling, bias, and batch effects. For this scope, we developed and optimized an automated plasma depletion pipeline composed of three major steps: sequential depletion, parallel digestion, and LC-MS acquisition (Figure 1A).

First, we automated the depletion of the 14 most abundant proteins using a sequential approach supporting a 96-well format.^48^ Briefly, after randomization and filtration of the samples into a 96-well plate, an automated chromatographic system sequentially and automatically processed the plate, thereby depleting the 14 most abundant human proteins in plasma via the use of specific antibodies.

To quantify the analytical gain of the approach and to assess whether depletion maintains quantitative precision and accuracy, we performed a controlled quantitative experiment (CQE). The CQE sample set was generated from 20 healthy human plasma samples spiked with either 1:400 *E. coli* and 1:90 *S. cerevisiae* for condition A or 1:200 *E. coli* and 1:120 *S. cerevisiae* for condition B (Figure 1B). After processing the 40 samples with or without the automated depletion pipeline, they were analyzed on a mass spectrometer using data-independent analysis (DIA). Since the major challenge linked to quantification in plasma is the large dynamic range, removing the 14 most abundant proteins should lead to an increase in the number of proteins identified compared to the neat plasma. Indeed, while the processing of the neat plasma samples led to an average identification of 572 proteins (3920 peptides) across all samples, depletion significantly increased the coverage by 257% to 1471 proteins (10,230 peptides) (*n* = 40, *p*-value = 1e – 98; Supporting Information Figure 1A). Importantly, depletion retained the quantitative accuracy close to the expected ratios between conditions B and A of 0.415 for *E. coli* and −1 for *S. cerevisiae*: *E. coli* median ratios −1.20 and −1.18 and *S. cerevisiae* 0.38 and 0.32 for the neat and depleted sets, respectively (Figure 1C). We observed a reduction in the intensity of the depleted proteins along with the closely related proteins (e.g., other immunoglobulins or apolipoproteins) while observing an overall increase in intensity in the rest of the plasma proteome (Supporting Information Figure 1B). Furthermore, intensities of human proteins are correlated between the two data sets (Pearson correlation 0.58, *n* = 247), and if only nondepleted proteins are considered, this correlation becomes much stronger (0.85, *n* = 198). Finally, we performed an unpaired *t*-test between conditions B and A and could identify 171 and 621 candidates (FDR, *q*-value ≥ 0.01) for the neat and depleted sets, respectively (Supporting Information Figure 1C). Given the experiment’s controlled nature, we could identify the true hits as those proteins mapping to either *E. coli* or *S. cerevisiae* and showing the expected directionality. Overall, the depletion led to a 362% increase in true hits, 170 and 615 for neat and depleted (actual FDR < 1% for both), respectively. In summary, the automated depletion more than tripled the number of proteins identified and the number of true hits while maintaining quantitative accuracy and reducing the manual workload to only the filtering of the samples (about half a day per 96 samples; Figure 1A).

In the second step following depletion, the sample plate was prepared for digestion on an automated platform using a protein aggregation capture approach.^36^ Subsequently, the samples were cleaned using C18 plates, and peptide concentration was measured. In case a library was generated, a fraction of all samples can be pooled and an ultra-high-pressure liquid chromatography-controlled high pH reverse-phase (HPRP) fractionation was performed.^38^

The third step comprises the LC-MS measurement of the samples. Even after depletion of the most abundant proteins, the major challenge hindering quantification is the large dynamic range in plasma. Hence, we developed and optimized the LC-MS acquisition for deep proteome coverage using FAIMS-based ion mobility on the orbitrap platform combined with high-performance chromatography. We developed FAIMS-DIA methods that maximize the protein and peptide identification by comparing values and counts of FAIMS compensation voltages with different scan resolutions. This resulted in a set of optimized methods for gradients from 1 to 4 h. Benchmarking with the depleted plasma resulted in 1300 protein identifications in 1 h gradients to 2103 protein identifications in 4 h (Figure 1D). For reference, in the human cell line HeLa, 10,026 proteins were identified in 4 h (Supporting Information Figure 1D).

Altogether, we demonstrated that the presented automated plasma depletion pipeline has the potential to enable the unbiased, reproducible, and precise quantification of more than 2000 proteins on average per sample across very large cohorts.

### Plasma Proteome Depth Achieved

**Figure 2:**
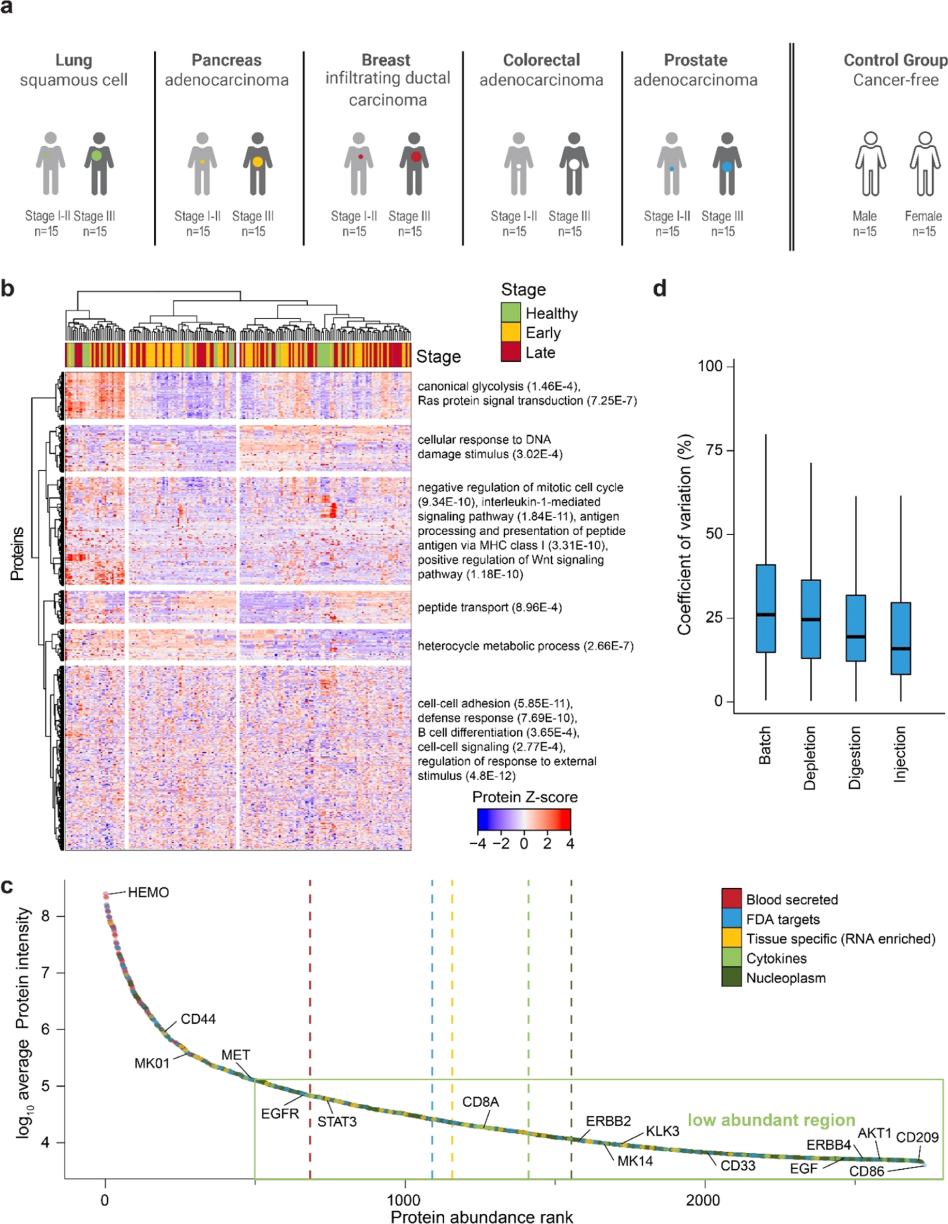
Deep plasma discovery proteomics of five solid cancer types. (a) Description of cohort comprising five solid cancers: breast (infiltrating ductal carcinoma), colon (adenocarcinoma), pancreas (adenocarcinoma), prostate (adenocarcinoma), and lung (non-small-cell lung cancer, squamous cell) cancers. Fifteen subjects for early and late stages were selected for each cancer type, along with 15 matching healthy individuals (a total of 30, given the need to balance ethnicity and sex for prostate and breast cancers). (b) *Z*-score of all quantified proteins (*n* = 2732) across all measured samples (*n* = 180). Stage calling is overlaid. Both the proteins and the samples were hierarchically clustered. Selected, significantly enriched gene ontology pathways are reported on the right with the *p*-value in parentheses. (c) The protein rank vs protein average intensity (*n* = 180). Proteins were categorized according to Human Protein Atlas, and the average rank was calculated (dotted, vertical lines). The green box depicts the proteome region that is typically below the sensitivity of the neat plasma profiling by mass spectrometry. (d) The coefficient of variation (CV) of the quality control measurements across the processing steps was plotted. The LC-MS variance was controlled by reinjection of the same digested sample (injection). Digestion and depletion were done repeatedly of the same sample (digest, depletion) and the batch stemming from sample preparation 96-well plates (batch). Thick lines indicate medians, boxes indicate 25 and 75% quartiles, and whiskers extend between the median and ±(1.58 × interquartile range).

To test our pipeline, we set out to analyze a diverse cohort of human plasma samples coming from the five most deadly solid cancer types in the United States:^35^ pancreatic, colorectal, breast, prostate, and non-small-cell lung cancers. For each cancer type, 15 early-stage (I–IIC) and 15 late-stage (IIIA–IIIC) nonmetastatic patients, as well as 15 matching normal control samples, were selected based on the available baseline data (including gender, age, and where applicable smoking status; Figure 2A and Supporting Information Table 2). Altogether, we processed 180 samples (and an additional 24 quality control samples) over the course of 1 week and approximately a month of measurement time. With this scalable approach, we could identify and quantify 2732 proteins (2463 proteins with two or more peptide sequences and on average with 9.2 peptides per protein) across 226 measurements (180 samples and 46 quality control samples, about 900 proteins/h measurement; Figure 2B), of which 1804 are found in at least 50% of the runs (Supporting Information Figure 2A). On average, we identified 1806 proteins per run. Importantly, missing values were stemming mostly from biological variation as in injection triplicates 88.7% of the 2209 protein groups detected are complete observations. Additionally, 77% of the 2402 protein groups from 15 injection replicates were complete, representing only 6% (119) less protein groups with full profiles than in injection triplicates. Across cancers (and the healthy cohort), the identifications varied between 2524 in prostate cancer and 2682 in lung cancer, showing that only a minimal part (<10%) of the identification is disease-specific and around 1000 protein groups are consistently quantified despite variable biology (Supporting Information Figure 2B). Furthermore, it can be assumed that peptides and proteins that do not fulfill the FDR criteria are below the limit of detection since DIA measures all ions and does not have the stochastic nature of DDA. With the identified proteins, we could cover the 8-order-of-magnitude dynamic range reported for plasma in the Human Protein Atlas (3222 proteins detected in human plasma by mass spectrometry, of which we could quantify 70%; Supporting Information Figure 2C). Within this range, we extensively covered the tissue leakage proteome, interleukins, and signaling proteins such as EGF, KLK3 (PSA), AKT1, CD86, MET, ERBB2, and CD33 (Figure 2C). As expected, among the 500 highest intensity proteins, meaning that proteins would likely be identified if no depletion would have been applied, 196 (39%) are classified as secreted proteins. On the lower end, we identified tissue-specific proteins coming from the diseased organs (*n* = 42, 81% of which are not part of the 500 most abundant proteins), cytokines (*n* = 29, 85%), and nucleoplasm (*n* = 637, 90%) proteins exemplifying different functional plasma concentration ranges (Figure 2C). We identified 190 targets for FDA-approved drugs, of which 125 (66%) fall in the lower intensity range.^49^ The different biological role of low and high abundant plasma proteins shows that we could recover the known biology of the plasma proteome.

Furthermore, based on quality control samples, we could characterize variance introduced on each level: injection (median coefficient of variation (CV = 16%), digestion (CV = 19%), depletion (CV = 25%), and column (CV = 26%)), all of which are much lower than the healthy interindividual variability (CV = 56%; Figure 2D and Supporting Information Figure 2D). As a further quality control, we focused on known protein levels’ interpatient variability (measured by CV; Supporting Information Figure 2E). On one hand, coagulation and complement cascade proteins (KEGG complement and coagulation cascades) were significantly enriched among the proteins with the least interpatient variability (median CV = 32% and *p*-value = 2.8e – 12), such as complement factor I (CF1, CV = 23%) and complement component C6 (CV = 27%), demonstrating tight regulation.^18^ On the other hand, keratins (likely contaminants, Go biological process keratinization) were significantly enriched among the proteins with the most interpatient variability (CV = 339% and *p*-value = 4.46e – 8), with HLA molecules (CV = 90%) also showing high variability across patients.^50^ Additionally, lipoprotein A (LPA) showcases a large interpatient variability (CV = 113%), likely due to the known genetic variants affecting its secretion into plasma.^51,52^ Overall, the quantitative data set generated recapitulates known biological features of intrapatient heterogeneity while providing a deep unbiased view of the plasma proteome.

### Considerable Heterogeneity across Cancer Types

**Figure 3:**
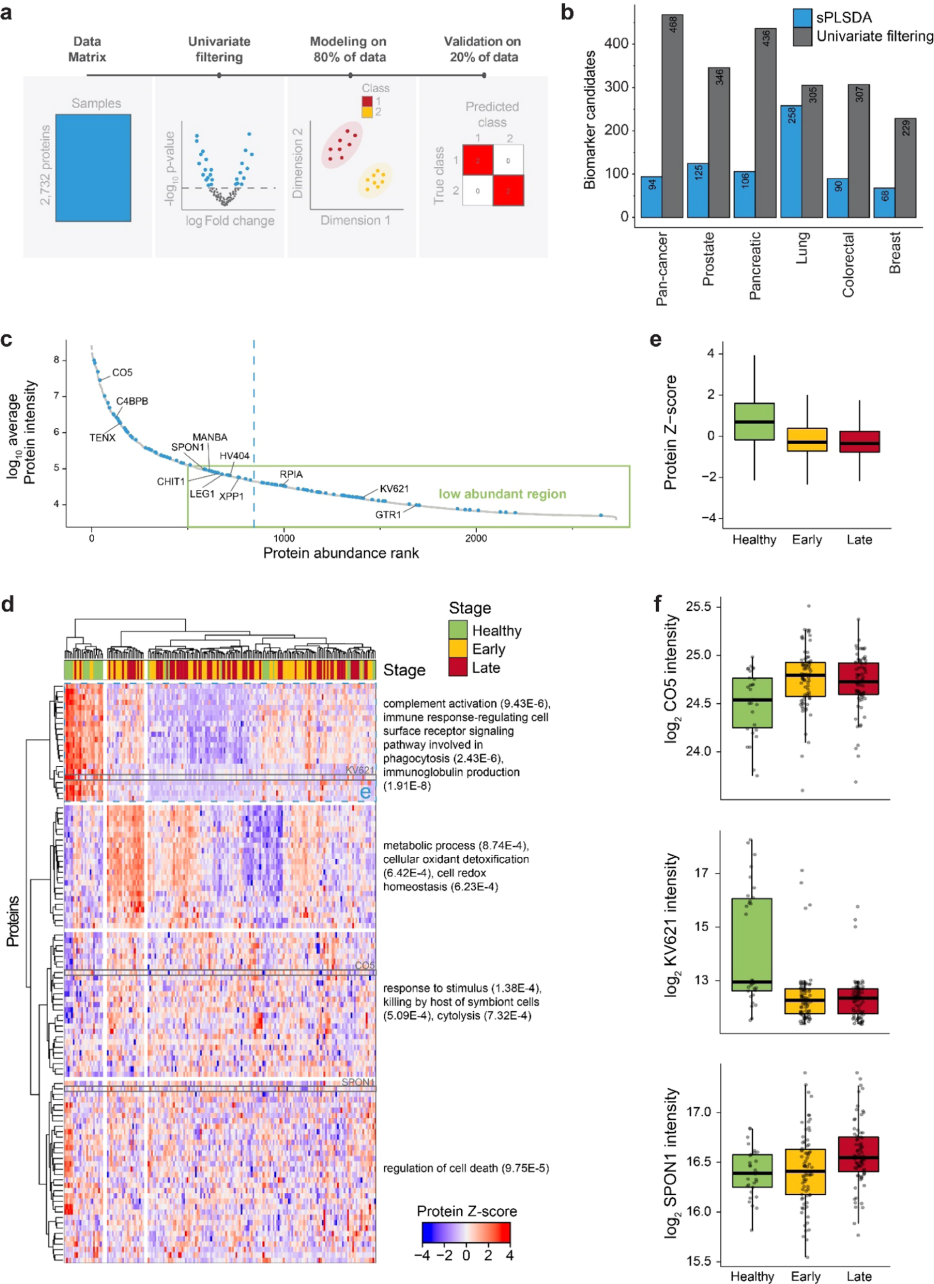
Machine learning-based candidate biomarker discovery. (a) Schematic detailing the steps of the postprocessing, including univariate testing for filtering, machine learning (sPLSDA) on 80% of the data, and classification performance accuracy on the 20% hold-out validation data. (b) Overview of the number of biomarker candidates selected by univariate analysis (gray) and machine learning (blue) for healthy, early, and late stages across all cancers and individual cancers. (c) Average protein intensity plotted vs protein abundance rank. The machine learning-selected biomarker candidates for the pan-cancer model are colored blue (the average is plotted as a blue line), and important contributors are highlighted. The green box depicts the proteome region that is typically below the sensitivity of neat plasma profiling by mass spectrometry. (d) *Z*-score of all machine learning-selected candidate biomarkers for the pan-cancer model (*n* = 94) across all measured samples (*n* = 180). Stage calling is overlaid. Both the proteins and the samples were hierarchically clustered. Selected, significantly enriched gene ontology pathways are reported on the right with the p-value in parentheses. Proteins highlighted in blue and gray are reported in panels (e) and (f), respectively. (e) Boxplot visualization of the average *z*-transformed protein intensity for all proteins (*n* = 288) in the cluster highlighted in blue in panel (d) divided by stage (*n* = 180). Thick lines indicate medians, boxes indicate 25 and 75% quartiles, and whiskers extend between the median and ±(1.58 × interquartile range). (f) Boxplot visualization (as in panel (e)) of the log-transformed protein quantities of the three most differentiating proteins based on the machine learning model (SPON1, KV621, and CO5). Each data point represents a sample (*n* = 180).

The cohort was designed to enable five independent within-cancer analyses, each comprising a healthy-, early-, and late-stage group (each *n* = 15; Supporting Information Table 2 and Figure 2A). Overall, we included 30 control samples, but only a subset of 15 per cancer were matched (see methods). Hence, a combined analysis of all samples together was not the primary goal of this study. Aware of these limitations, we explored the entire data set for markers that would agnostically predict the cancer stage. The analysis pipeline applied to the whole data set, and the cancer-specific analyses were the same and aimed at providing actionable insights about specific disease development. Given a large amount of data (2732 proteins combined), we performed a two-step approach (Figure 3A). First, we filtered for differentially abundant proteins between healthy-, early- and late-stage cancers using univariate analysis. In the case of the pan-cancer model, we found 468 proteins dysregulated (Figure 3B, Supporting Information Figure 3A, and Supporting Information Table 3). Second, using the selected proteins, we trained a model based on sparse partial least-squares discriminant analysis (sPLSDA) on 80% of the data set. This modeling step further reduced the number of proteins to 94 (Figure 3B). The model partially differentiated healthy from disease but not late to early stage (Supporting Information Figure 3B and Supporting Information Table 4). Interestingly, the majority of the differentiating proteins would have been below the detection level in a neat plasma preparation (65%; Figure 3C). Furthermore, the unsupervised clustering of the differentiating proteins generated enriched patterns (Figure 3D). For example, proteins enriched for immunoglobulin production and complement activation tend to be higher in healthy samples (Figure 3E). A subset of cancer samples have a strong upregulation of proteins linked to metabolic processes and cellular oxidant detoxification (Figure 3D,E). Immunoglobulin kappa variable 6–21 (KV621) was among the proteins higher in healthy samples, was the third most important discriminant protein in the model (0.56 importance), and showed a more pronounced bimodal distribution in healthy individuals and a decrease in diseased individuals (Figure 3F and Supporting Information Figure 3C). In addition, the model identified the known inflammation marker complement C5 (CO5, importance 1^53^) increased in the early and late stages and spondin-1 (SPON1, importance 0.58) increased in the late stage (Figure 3F and Supporting Information Figure 3C), as the first and second most important contributors, respectively. Finally, the predictive power of the model was validated using the remaining 20% of the samples. The predictive power was low at 55.6% (Supporting Information Figure 3D), likely due to the cohort imbalance, the sample heterogeneity, and the small sample set, as each cancer type is known to have a particular protein signature.^54^ Nonetheless, unsupervised clustering using the final protein panel (enrichment *p*-value = 1.4e – 9) allowed for a more efficient separation of samples between healthy and diseased states compared to the entire proteome (*p*-value = 0.09; Figures 2B and 3D). Altogether, the global data analysis underlined the importance and necessity of precision medicine and a much larger sample set would be needed to find a potential “one-fits-all” solution.

### Overall Changes within and across Cancer Types

**Figure 4:**
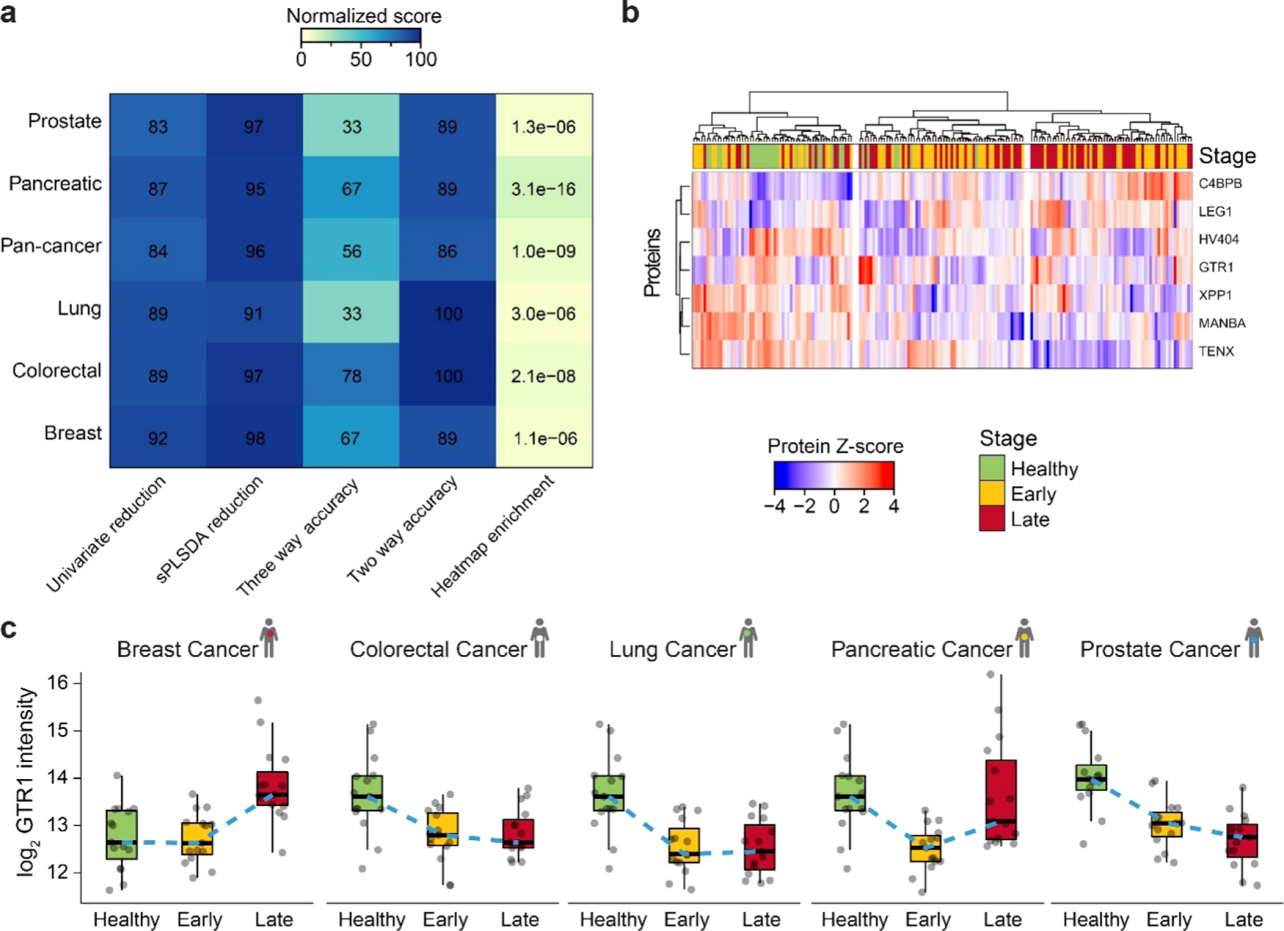
Classification accuracy of the five cancer types. (a) Overview of the data analysis per cancer and combined (pan-cancer) as a normalized score. Percentage reduction upon univariate filtering and sPLSDA on 80% of the data set along with percentage accuracy as measured on the 20% hold-out samples as a three-way (healthy-, early-, and late-stage) and two-way (cancer and healthy) classification and p-value of enrichment based on the heatmap clustering (Manhattan distance, Ward clustering). (b) *Z*-score of the seven candidate proteins consistently selected across all cancers (by univariate analysis, *n* = 180). Stage calling is overlaid. Both the proteins and the samples were hierarchically clustered. (c) Boxplot visualization of log-transformed GTR1 quantities across the stage and cancer type. The healthy samples were matched to the respective cancer samples. Thick lines indicate medians, boxes indicate 25 and 75% quartiles, whiskers extend between the median and ±(1.58 × interquartile range), and each data point represents a sample (*n* = 180). The dashed blue line connects the median values across stages.

Next, we applied the same analysis strategy using the matched healthy controls to each of the five solid tumor types. In the first step, we identified on average 325 significantly altered proteins between healthy, late, and early stages (Figures 3B and 4A and Supporting Information Table 3). With 436 significantly altered proteins (83% reduction in features), prostate cancer had the highest number of differentially abundant proteins, while breast cancer had the fewest with 229 (92% reduction). Interestingly, only a few proteins were shared among cancers (Supporting Information Figure 4A). Pancreatic and prostate had the most with 190 overlapping proteins, while breast and pancreas had the least at 37 (Supporting Information Figure 4A). Seven candidate proteins were consistently selected as differentially abundant across all cancers: the complement activation protein C4b-binding protein β chain (C4BPB), the immunoglobulin component immunoglobulin heavy variable 4-4 (HV404), the T-cell apoptosis inducer galectin-1 (LEG1), the degrader of the inflammation-promoting bradykinin peptide Xaa-Pro aminopeptidase 1 (XPP1), the solute carrier family 2 facilitated glucose transporter member 1 (GTR1), the glycan metabolism β-mannosidase enzyme (MANBA), and the suggested growth inducer of epithelial tumors tenascin-X (TENX; Figure 4B and Supporting Information Figure 4A,B). These candidates have rather decreasing (HV404, XPP1, MANBA, TENX) or increasing (LEG1, C4BPB) trends in a cancer agnostic manner, with the exception of GTR1, which strongly increases in the late-stage breast cancer while decreasing in the other types (Figure 4C). Interestingly, this small set of proteins separated healthy- from the cancer-stage samples quite well (*p*-value = 1.9e – 8; Figure 4B). Fitting an sPLSDA model with 80% of the data overall decreased the number of candidates to less than 5% of the total measured proteins. It led to an average of 129 candidates, making biological interpretation and follow-up more feasible (Figures 3B and 4A and Supporting Information Table 4). The relative decrease in the input data was highly cancer-dependent, from an almost 76% reduction in pancreatic cancer to only a 15% reduction in lung cancer. The number of overlapping proteins across models was minimal, likely due to the reductionist approach of sPLSDA and cancer-type-specific mechanisms, with no proteins being selected for all models (Supporting Information Figure 4C). Still, TAGL and MANBA were selected in all but breast cancer models, and GTR1 and LEG10 in all but the pan-cancer and breast cancer models (Figure 4C and Supporting Information Figure 4B).

In summary, the model classification performance measured on the 20% validation set ranged between 33.3% in lung and prostate cancers and 77.8% in colorectal cancer when all three groups were considered and between 86.1% for the pan-cancer model and 100% for lung and colorectal cancers when healthy and overall disease status were considered (Figure 4A and Supporting Information Table 2). While for the early/late-stage differentiation two of the six models were close to random performance, the disease status was easier to predict, especially if the cancer type is known, as the pan-cancer model performed the worst with an 86% accuracy. Interestingly, high model performance was not always associated with high separation efficiency using PCA or distance analysis and vice versa (Figure 4A). This is especially apparent in the case of pancreatic and colorectal cancers. While colorectal performs the best on the validation set, especially in the differentiation of healthy/disease, pancreatic cancer leads to the best separation by hierarchical clustering on all three groups (*p*-value = 3.1e – 16). In a nutshell, in contrast to the “one-fits-all” approach, the cancer-specific models performed better. In some cases, the classification accuracy of the derived models was good, demonstrating the benefit of deep profiling of the plasma proteome.

### Diseased State Separation in Colorectal Cancer

**Figure 5:**
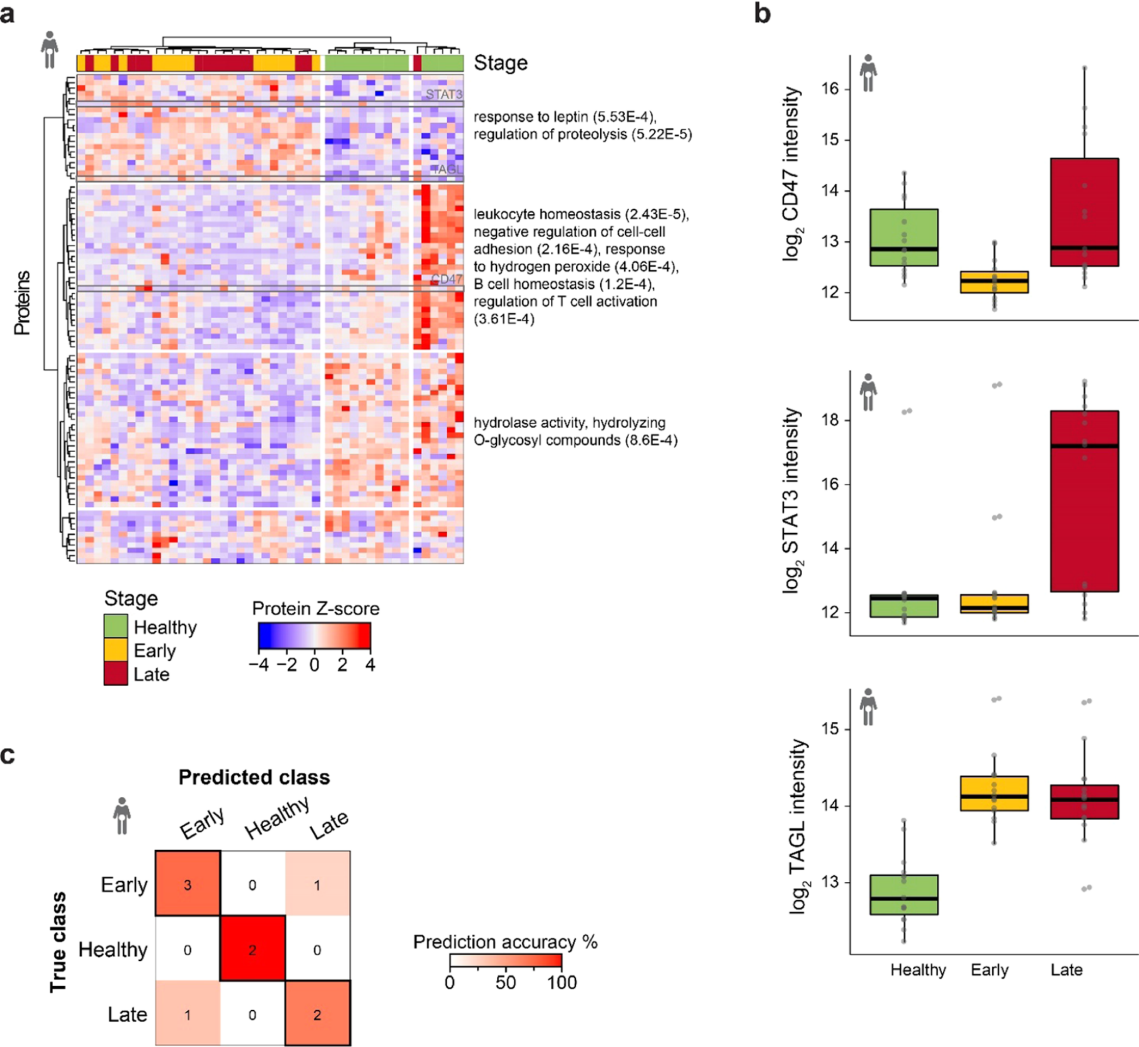
Colorectal cancer biomarker candidates predict diseased status. (a) *Z*-score of all machine learning-selected candidate biomarkers for the colorectal cancer model (*n* = 90) across the matched colorectal sample set (*n* = 45). Stage calling is overlaid. Both the proteins and the samples were hierarchically clustered. Selected, significantly enriched gene ontology pathways are reported on the right with the *p*-value in parentheses. Proteins highlighted in gray are reported in panel (b). (b) Boxplot visualization of log-transformed CD47, STAT3, and TAGL quantities divided by the stage for the colorectal cancer set. Thick lines indicate medians, boxes indicate 25 and 75% quartiles, whiskers extend between the median and ±(1.58 × interquartile range), and each data point represents a sample (*n* = 45). (c) Overview of the classification accuracy of the machine learning models for the colorectal cancer validation set (*n* = 9). Correct classifications are represented in the highlighted boxes.

In colorectal cancer (CRC), we identified 307 proteins significantly altered between healthy, early, and late stages (Supporting Information Figure 5A). The sPLSDA model further reduced these candidate proteins to 90, and both hierarchical clustering and PCA analysis led to the efficient separation of healthy subjects from patients regardless of tumor staging (*p*-value = 2.1e – 8; Figure 5A and Supporting Information Figure 5B). Multiple biological GO enrichments in the candidates could be dissected, for example, response to leptin and regulation of proteolysis increased in cancer (including STAT3 and transgelin (TAGL)). In contrast, the negative regulation of cell–cell adhesion, leukocyte homeostasis, and response to hydrogen peroxide decreased (including CD47; Figure 5A,B). TAGL (importance = 1.00), STAT3 (importance = 0.65), and CD47 (importance = 0.57) were the three most predictive proteins from the sPLSDA model and showed interesting patterns (Figure 5B and Supporting Information Figure 5C). While CD47 and STAT3 showed strong heterogeneity in late-stage colorectal cancer, TAGL was highly expressed in the early- and late-stage colorectal cancers (Figure 5B). The selected 90 proteins were distributed across the entire intensity range of measured proteins, with more than 80% of the selected proteins (including the three most important) being beyond the 500 protein mark representing the usual range of proteins detected in neat plasma (Supporting Information Figure 5D). Furthermore, at 78%, the model had the best overall classification accuracy among all tested malignancies on the validation set (Figure 5C). As no misclassification for healthy subjects was observed, the panel of identified candidate proteins could be helpful for early CRC diagnosis. In summary, despite the small sample set, deep profiling of the human plasma enabled the partial classification of diseased patients based on a panel of 90 proteins that span a large dynamic range while providing an unbiased glimpse into the biological processes at the base of colorectal cancer.

### Stage Separation in Pancreatic Cancer

**Figure 6:**
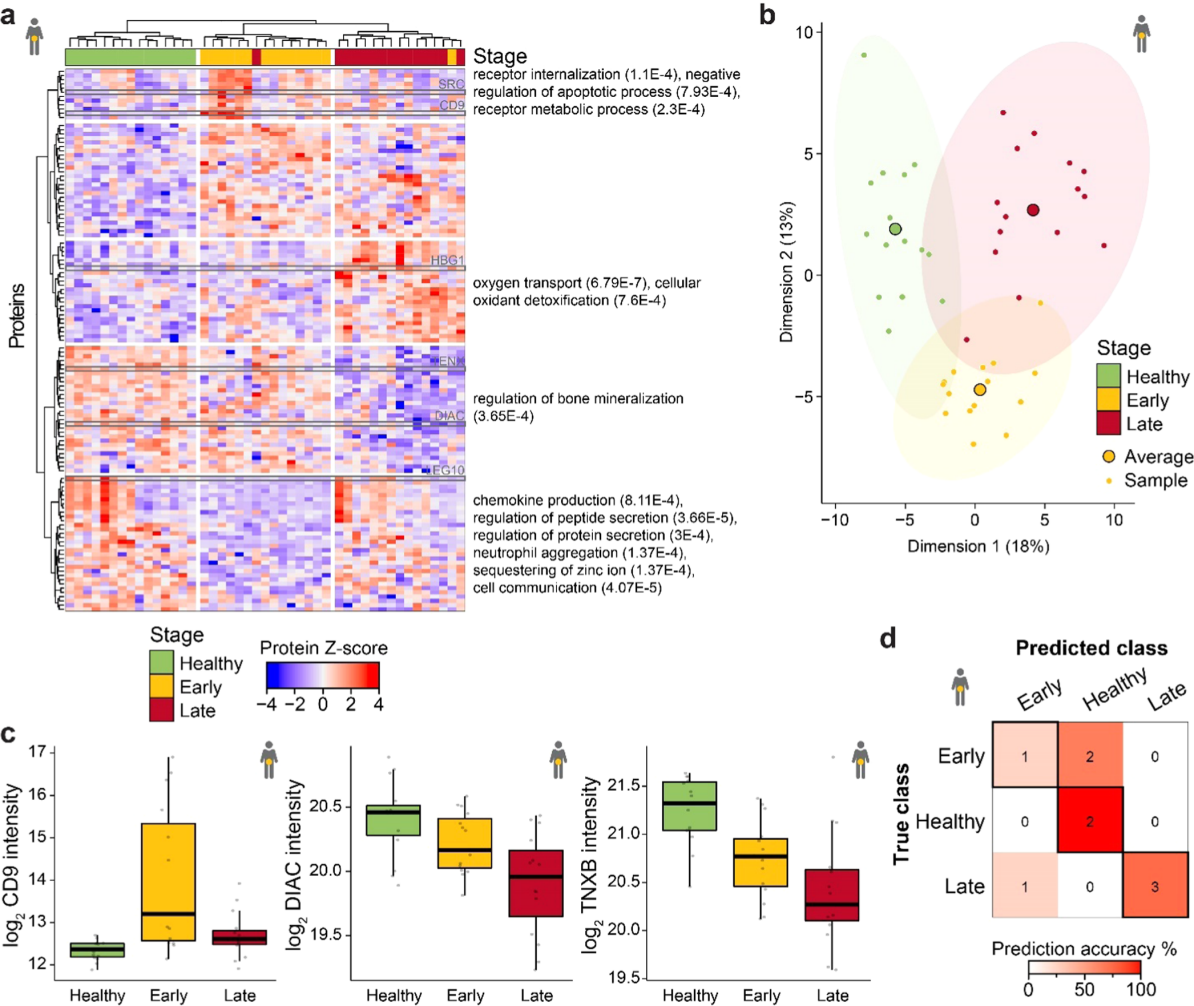
Pancreatic cancer biomarker candidates predict diseased stage. (a) *Z*-score of all machine learning-selected candidate biomarkers for the pancreatic cancer model (*n* = 106) across the matched pancreatic cancer sample set (*n* = 45). Stage calling is overlaid. Both the proteins and the samples were hierarchically clustered. Selected, significantly enriched gene ontology pathways are reported on the right with the p-value in parentheses. Proteins highlighted in gray are reported in panel (c) and the Supporting Information Figure 6. (b) Representation of the first two dimensions from the PCA analysis based on candidates identified in the sPLSDA model for pancreatic cancer. Small points represent samples, and large points represent the average across the stage. While the first dimension separates healthy from diseased samples and explains 18% of the variance in the data, the second dimension separates early- and late-stage samples and represents 13% of the variability. The corresponding ellipses represent sample concentration around the mean. (c) Boxplot visualization of log-transformed CD9, DIAC, and TNXB quantities divided by the stage for the pancreatic cancer set. Thick lines indicate medians, boxes indicate 25 and 75% quartiles, whiskers extend between the median and ± (1.58 × interquartile range), and each data point represents a sample (*n* = 45). (d) Overview of the classification accuracy of the machine learning models for the pancreatic cancer validation set (*n* = 9). Correct classifications are represented in the highlighted boxes.

In the pancreatic cancer set, 436 proteins were significantly altered between healthy, early, and late stages (Supporting Information Figure 6A). The sPLSDA modeling selected 106 proteins, which efficiently separated the three classes in both hierarchical clustering and PCA analyses (*p*-value = 3.1e – 16; Figure 6A,B). The separation was driven primarily by CD9 (importance = 0.37), TENX (importance = 0.32), and di-N-acetylchitobiase (DIAC, importance = 0.28), with both TENX and DIAC showing a downregulation with disease progression and CD9 showing a stronger upregulation in early- than late-stage pancreatic cancer (Figure 6C and Supporting Information Figure 6B). CD9 levels correlated most strongly with endocytosis-related protein dynamin-1 (DYN1), heat shock protein β-1 (HSPB1), platelet glycoprotein 4 (CD36), and a profibrotic matricellular protein CCN family member 2 (CCN2). The unsupervised clustering of the candidate proteins resulted in interesting patterns (Figure 6A). In the early-stage pancreatic cancer, proteins involved in the regulation of peptide secretion, cell communication, and chemokine production are overall downregulated including LEG10, which is essential for the suppressive function of CD25 positive regulatory T-cells^55,56^ (Supporting Information Figure 6C), while proteins involved in the negative regulation of apoptotic process and receptor internalization (including proto-oncogene tyrosine-protein kinase Src (SRC) and CD9; Figure 6C and Supporting Information Figure 6C) are upregulated. In late-stage pancreatic cancer, cellular oxidant detoxification and oxygen transport, including hemoglobin subunit γ-1 (HBG1), are upregulated (Supporting Information Figure 6C). Of the 125 biomarker candidates selected, 65% were in the low abundance range (Supporting Information Figure 6D). In the validation set, the model had an accuracy of 66.7%, with two out of nine observations incorrectly assigned to the healthy group instead of early-stage cancer (Figure 6D). On the whole, deep profiling of human plasma enabled the clustering of diseased patients based on the disease stage and feature reduction makes biological patterns related to disease progression emerge.

## Discussion

We have developed an automated, robust, and parallelizable workflow for deep, large-scale plasma proteome profiling by depletion and sample preparation and by generating deep coverage ion mobility DIA methods. First, we demonstrated substantial improvements upon depletion for identification and quantification using a controlled quantitative plasma experiment. Furthermore, through multistage quality control, we assessed the variance introduced at each step of processing. In summary, the novel plasma discovery workflow enables the deep profiling of 10 samples per day per analytical platform to a depth of approximately 2700 proteins per study for 2 h gradients, reaching deep into tissue leakage and signaling molecules while maintaining quantitative accuracy. To evaluate the potential of deeper proteome coverage of the analytical pipeline, we measured a subset of the cancer plasma study with 3.5 h gradient FAIMS-DIA acquisitions. This resulted in a substantial increase in protein identifications to 3372 cumulatively (Supporting Information Figure 7).

Next, we applied the novel plasma discovery workflow to a cohort containing samples coming from five solid tumors. Data analysis, including machine learning, revealed biomarker candidates and resulted in predictive models. The biomarkers mainly contain proteins from low abundance regions that would have likely been missed by neat plasma profiling, as previously speculated by Geyer et al.^9^ Given the limited sample size and sample selection limitations (e.g., the healthy samples are self-declared healthy), the presented biomarker candidates require additional validation in an independent cohort.

While the separation of healthy from cancer plasma samples was quite accurate for the cancer-specific models (average accuracy 93%), early- to late-stage differentiation was much more challenging, showing weaker separation (average accuracy 56%). The pan-cancer model performed worse than the cancer-specific models, indicating that “one-fits-all” biomarkers are generally harder to discover. This is likely because of the considerable heterogeneity across cancer types and could be solved by a larger cohort, more advanced stratification strategy and would likely lead to a larger biomarker panel.

Seven candidate proteins were consistently differentially abundant across all cancers, of which one followed a cancer-type-specific behavior. Notably, the previously reported pan-cancer biomarker candidate TENX was reproduced, showing a reduction in the disease progression irrespective of the cancer type.^57^ Overall, our approach showed that deep exploration of the proteome of cancer plasma samples can be realized for biomarker discovery. Larger cohorts and a longitudinal study design, where the same subjects are monitored ideally before disease onset, would likely lead to more robust biomarkers.

When focusing on colorectal cancer, 307 proteins were altered between healthy, early, and late stages. These include three with a documented role in colorectal cancer development: STAT3,^58^ TAGL,^59^ and CD47.^60^ In addition, gene ontology enrichments based on identified candidates showed a response to leptin and the regulation of proteolysis increased in cancer. At the same time, there was a negative regulation of cell–cell adhesion, leukocyte homeostasis, and response to hydrogen peroxide. Based on the machine learning-assisted biomarker discovery approach, a prediction model based on 90 proteins had the highest predictive classification power with a 78% accuracy on the hold-out set.

In pancreatic cancer, 436 proteins were altered between healthy, early, and late stages. Of these, seven (GTR1, APOA4, IBP2, CD9, CAB45, OLFM4, BGH3) have previously been suggested as possible pancreatic cancer biomarkers.^61−65^ Machine learning-based modeling selected 106 proteins, which led to an efficient separation using distance measures of healthy-, early-, and late-stage samples. The selected proteins showed an average overall prediction accuracy of 67%, with two observations incorrectly assigned to the healthy group instead of early-stage cancer. This separation was primarily driven by the three cancer-related proteins CD9,^66^ TENX,^57^ and DIAC.^62,67^ Further proving the quality of the candidates, the separation was also driven by the recently proposed therapeutic target CNN2^68^ and the prognostic marker GTR1.^69^ A study by Jayaraman et al. demonstrated that the exposure of pancreatic cancer cells to zinc leads to increased protein ubiquitination and enhanced cell death, implicating zinc as a potential therapy in treating pancreatic cancer.^70^ We found the sequestration of zinc ions as an enriched biological process in pancreatic cancer, specifically downregulated in cancer samples (especially early stage).

Clinical analysis of blood is the most widespread diagnostic procedure in medicine, and blood biomarkers are used to diagnose diseases, categorize patients, and support treatment decisions. The presented approach is well suited for deep, epidemiological biomarker studies in plasma as it reaches deep into the tissue leakage area, where information on the health state of distal tissues can be discovered. Furthermore, biomarker sets derived from the machine learning biomarker discovery analysis are not optimally suited for a direct transition into a “classical” clinical biomarker, as new multiplexed approaches for clinical assays would be required. Such challenges could potentially be facilitated by DIA or multiple PRM-based assays, which are fully compatible with the presented workflow and could ultimately result in streamlined discovery-to-target-driven personalized medicine utilizing only one technology platform.^71,72^

Hence, we envision that the profiling of large cohorts at high proteome depth will strongly support the development of novel biomarkers previously not accessible to large-scale discovery approaches and will lead to the development of biomarker panels that will finally deliver on the promise of noninvasive, preventive cancer screening.

## Supporting Information Available

Controlled quantitative experiment with plasma and mass spectrometric performance benchmarking (Figure S1); deep plasma discovery proteomics of five solid cancer types (Figure S2); pan-cancer predictive model based on deeply profiled plasma (Figure S3); biomarker candidates within and across the five solid cancers (Figure S4); colorectal cancer analysis (Figure S5); pancreatic cancer analysis (Figure S6); identifications for the cancer plasma study in the dependence of gradient length (Figure S7) (PDF)
Sample origin table (Table S1) (XLS)
Cancer cohort composition table (Table S2) (XLS)
List of the differentially abundant proteins between healthy-, early-, and late-stage cancers using univariate analysis (Table S3) (XLSX)
List of the proteins selected by sPLSDA for each cancer type along with their importance (Table S4) (XLSX)

## Notes

The MS data, the spectral libraries, and the quantitative data tables have been deposited to the ProteomeXchange Consortium via the MassIVE repository^73^ with the data set identifier MSV000088180. The saved projects from Spectronaut can be viewed with the Spectronaut Viewer (www.biognosys.com/spectronaut-viewer).

## Notes

The authors R.B., M.T., K.S., D.K., J.M., S.M., and L.R. are full-time employees of Biognosys AG (Schlieren-Zurich, Switzerland). Spectronaut is a trademark of Biognosys AG.

## References

[ref1] Murphy R. M.; Tsai A. M. Misbehaving Proteins, Springer: New York: New York, NY, 2006.

[ref2] Végvári Á.; Welinder C.; Lindberg H.; Fehniger T. E.; Marko-Varga G. Biobank Resources for Future Patient Care: Developments, Principles and Concepts. J. Clin. Bioinf. 2011, 1, 24 10.1186/2043-9113-1-24. PMC3197484 21923917

[ref3] American Diabetes Association Diagnosis and Classification of Diabetes Mellitus. Diabetes Care 2013, 36, S67–S74. 10.2337/dc13-S067. 23264425 PMC3537273

[ref4] Burla B.; Arita M.; Arita M.; Bendt A. K.; Cazenave-Gassiot A.; Dennis E. A.; Ekroos K.; Han X.; Ikeda K.; Liebisch G.; Lin M. K.; Loh T. P.; Meikle P. J.; Orešič M.; Quehenberger O.; Shevchenko A.; Torta F.; Wakelam M. J. O.; Wheelock C. E.; Wenk M. R. MS-Based Lipidomics of Human Blood Plasma: A Community-Initiated Position Paper to Develop Accepted Guidelines. J. Lipid Res. 2018, 59, 2001–2017. 10.1194/JLR.S087163. 30115755 PMC6168311

[ref5] Tsui N. B. Y.; Ng E. K. O.; Lo Y. M. D. Molecular Analysis of Circulating RNA in Plasma. Methods Mol. Biol. 2006, 336, 123–134. 10.1385/1-59745-074-X:123. 16916258

[ref6] Cescon D. W.; Bratman S. V.; Chan S. M.; Siu L. L. Circulating Tumor DNA and Liquid Biopsy in Oncology. Nat. Cancer 2020, 1, 276–290. 10.1038/s43018-020-0043-5. 35122035

[ref7] Cohen J. D.; Li L.; Wang Y.; Thoburn C.; Afsari B.; Danilova L.; Douville C.; Javed A. A.; Wong F.; Mattox A.; Hruban R. H.; Wolfgang C. L.; Goggins M. G.; Dal Molin M.; Wang T.-L.; Roden R.; Klein A. P.; Ptak J.; Dobbyn L.; Schaefer J.; Silliman N.; Popoli M.; Vogelstein J. T.; Browne J. D.; Schoen R. E.; Brand R. E.; Tie J.; Gibbs P.; Wong H.-L.; Mansfield A. S.; Jen J.; Hanash S. M.; Falconi M.; Allen P. J.; Zhou S.; Bettegowda C.; Diaz L. A.; Tomasetti C.; Kinzler K. W.; Vogelstein B.; Lennon A. M.; Papadopoulos N. Detection and Localization of Surgically Resectable Cancers with a Multi-Analyte Blood Test. Science 2018, 359, 926–930. 10.1126/science.aar3247. 29348365 PMC6080308

[ref8] Rappaport N.; Twik M.; Plaschkes I.; Nudel R.; Stein T. I.; Levitt J.; Gershoni M.; Morrey C. P.; Safran M.; Lancet D. MalaCards: An Amalgamated Human Disease Compendium with Diverse Clinical and Genetic Annotation and Structured Search. Nucleic Acids Res. 2017, 45, D877–D887. 10.1093/NAR/GKW1012. 27899610 PMC5210521

[ref9] Hernández B.; Parnell A.; Pennington S. R. Why Have so Few Proteomic Biomarkers “Survived” Validation? (Sample Size and Independent Validation Considerations). Proteomics 2014, 14, 1587–1592. 10.1002/PMIC.201300377. 24737731

[ref10] Orton D. J.; Doucette A. A. Proteomic Workflows for Biomarker Identification Using Mass Spectrometry — Technical and Statistical Considerations during Initial Discovery. Proteomes 2013, 1, 109 10.3390/PROTEOMES1020109. 28250400 PMC5302744

[ref11] Drucker E.; Krapfenbauer K. Pitfalls and Limitations in Translation from Biomarker Discovery to Clinical Utility in Predictive and Personalised Medicine. EPMA J. 2013, 4, 7 10.1186/1878-5085-4-7. 23442211 PMC3599714

[ref12] Ignjatovic V.; Geyer P. E.; Palaniappan K. K.; Chaaban J. E.; Omenn G. S.; Baker M. S.; Deutsch E. W.; Schwenk J. M. Mass Spectrometry-Based Plasma Proteomics: Considerations from Sample Collection to Achieving Translational Data. J. Proteome Res. 2019, 18, 4085–4097. 10.1021/ACS.JPROTEOME.9B00503. 31573204 PMC6898750

[ref13] Anderson N. L.; Anderson N. G. The Human Plasma Proteome: History, Character, and Diagnostic Prospects. In Molecular & Cellular Proteomics: MCP., American Society for Biochemistry and Molecular Biology, 2002; pp 845–867. 10.1074/mcp.r200007-mcp200 12488461

[ref14] Skates S. J.; Gillette M. A.; LaBaer J.; Carr S. A.; Anderson L.; Liebler D. C.; Ransohoff D.; Rifai N.; Kondratovich M.; Težak Ž.; Mansfield E.; Oberg A. L.; Wright I.; Barnes G.; Gail M.; Mesri M.; Kinsinger C. R.; Rodriguez H.; Boja E. S. Statistical Design for Biospecimen Cohort Size in Proteomics-Based Biomarker Discovery and Verification Studies. J. Proteome Res. 2013, 12, 5383–5394. 10.1021/PR400132J. 24063748 PMC4039197

[ref15] Geyer P. E.; Kulak N. A.; Pichler G.; Holdt L. M.; Teupser D.; Mann M. Plasma Proteome Profiling to Assess Human Health and Disease. Cell Syst. 2016, 2, 185–195. 10.1016/j.cels.2016.02.015. 27135364

[ref16] Geyer P. E.; Holdt L. M.; Teupser D.; Mann M. Revisiting Biomarker Discovery by Plasma Proteomics. Mol. Syst. Biol. 2017, 13, 942 10.15252/msb.20156297. 28951502 PMC5615924

[ref17] Liu Y.; Buil A.; Collins B. C.; Gillet L. C.; Blum L. C.; Cheng L.; Vitek O.; Mouritsen J.; Lachance G.; Spector T. D.; Dermitzakis E. T.; Aebersold R. Quantitative Variability of 342 Plasma Proteins in a Human Twin Population. Mol. Syst. Biol. 2015, 11, 786 10.15252/MSB.20145728. 25652787 PMC4358658

[ref18] Cominetti O.; Núñez Galindo A.; Corthésy J.; Oller Moreno S.; Irincheeva I.; Valsesia A.; Astrup A.; Saris W. H. M.; Hager J.; Kussmann M.; Dayon L. Proteomic Biomarker Discovery in 1000 Human Plasma Samples with Mass Spectrometry. J. Proteome Res. 2016, 15, 389–399. 10.1021/acs.jproteome.5b00901. 26620284

[ref19] Bruderer R.; Muntel J.; Müller S.; Bernhardt O. M.; Gandhi T.; Cominetti O.; Macron C.; Carayol J.; Rinner O.; Astrup A.; Saris W. H. M.; Hager J.; Valsesia A.; Dayon L.; Reiter L. Analysis of 1508 Plasma Samples by Capillary-Flow Data-Independent Acquisition Profiles Proteomics of Weight Loss and Maintenance. Mol. Cell. Proteomics 2019, 18, 1242–1254. 10.1074/MCP.RA118.001288. 30948622 PMC6553938

[ref20] Messner C. B.; Demichev V.; Wendisch D.; Michalick L.; White M.; Freiwald A.; Textoris-Taube K.; Vernardis S. I.; Egger A. S.; Kreidl M.; Ludwig D.; Kilian C.; Agostini F.; Zelezniak A.; Thibeault C.; Pfeiffer M.; Hippenstiel S.; Hocke A.; von Kalle C.; Campbell A.; Hayward C.; Porteous D. J.; Marioni R. E.; Langenberg C.; Lilley K. S.; Kuebler W. M.; Mülleder M.; Drosten C.; Suttorp N.; Witzenrath M.; Kurth F.; Sander L. E.; Ralser M. Ultra-High-Throughput Clinical Proteomics Reveals Classifiers of COVID-19 Infection. Cell Syst. 2020, 11, 11–24.e4. 10.1016/J.CELS.2020.05.012. 32619549 PMC7264033

[ref21] Lee P. Y.; Osman J.; Low T. Y.; Jamal R. Plasma/Serum Proteomics: Depletion Strategies for Reducing High-Abundance Proteins for Biomarker Discovery. Bioanalysis 2019, 11, 1799–1812. 10.4155/BIO-2019-0145. 31617391

[ref22] Cao X.; Sandberg A.; Araújo J. E.; Cvetkovski F.; Berglund E.; Eriksson L. E.; Pernemalm M. Evaluation of Spin Columns for Human Plasma Depletion to Facilitate MS-Based Proteomics Analysis of Plasma. J. Proteome Res. 2021, 20, 4610–4620. 10.1021/acs.jproteome.1c00378. 34320313 PMC8419864

[ref23] Kaur G.; Poljak A.; Ali S. A.; Zhong L.; Raftery M. J.; Sachdev P. Extending the Depth of Human Plasma Proteome Coverage Using Simple Fractionation Techniques. J. Proteome Res. 2021, 20, 1261–1279. 10.1021/acs.jproteome.0c00670. 33471535

[ref24] Duffy M. J. Tumor Markers in Clinical Practice: A Review Focusing on Common Solid Cancers. Med. Princ. Pract. 2013, 22, 4–11. 10.1159/000338393. 22584792 PMC5586699

[ref25] Zhang X.; Xiao Z.; Liu X.; Du L.; Wang L.; Wang S.; Zheng N.; Zheng G.; Li W.; Zhang X.; Dong Z.; Zhuang X.; Wang C. The Potential Role of ORM2 in the Development of Colorectal Cancer. PLoS One 2012, 7, e31868 10.1371/journal.pone.0031868. 22363757 PMC3283705

[ref26] Gao F.; Zhang X.; Whang S.; Zheng C. Prognostic Impact of Plasma ORM2 Levels in Patients with Stage II Colorectal Cancer. Ann. Clin. Lab. Sci. 2014, 44, 388–393. 25361921

[ref27] Enroth S.; Berggrund M.; Lycke M.; Broberg J.; Lundberg M.; Assarsson E.; Olovsson M.; Stålberg K.; Sundfeldt K.; Gyllensten U. High Throughput Proteomics Identifies a High-Accuracy 11 Plasma Protein Biomarker Signature for Ovarian Cancer. Commun. Biol. 2019, 2, 221 10.1038/s42003-019-0464-9. 31240259 PMC6586828

[ref28] Chang T.-T.; Ho C.-H. Plasma Proteome Atlas for Differentiating Tumor Stage and Post-Surgical Prognosis of Hepatocellular Carcinoma and Cholangiocarcinoma. PLoS One 2020, 15, e0238251 10.1371/journal.pone.0238251. 32845921 PMC7449477

[ref29] Zhou B.; Zhou Z.; Chen Y.; Deng H.; Cai Y.; Rao X.; Yin Y.; Rong L. Plasma Proteomics-Based Identification of Novel Biomarkers in Early Gastric Cancer. Clin. Biochem. 2020, 76, 5–10. 10.1016/J.CLINBIOCHEM.2019.11.001. 31765635

[ref30] Klocker H.; Golding B.; Weber S.; Steiner; Eberhard; Tennstedt P.; Keller; Thomas; Schiess R.; Gillessen S.; Horninger W.; Steuber T. Development and Validation of a Novel Multivariate Risk Score to Guide Biopsy Decision for the Diagnosis of Clinically Significant Prostate Cancer. BJUI Compass 2020, 1, 15–20. 10.1002/BCO2.8. 35474911 PMC8988838

[ref31] Rai A. J.; Zhang Z.; Rosenzweig J.; Shih L. ming.; Pham T.; Fung E. T.; Sokoll L. J.; Chan D. W. Proteomic Approaches to Tumor Marker Discovery. Arch. Pathol. Lab. Med. 2002, 126, 1518–1526. 10.5858/2002-126-1518-PATTMD. 12456215

[ref32] Zhang Z.; Bast R. C.; Yu Y.; Li J.; Sokoll L. J.; Rai A. J.; Rosenzweig J. M.; Cameron B.; Wang Y. Y.; Meng X. Y.; Berchuck A.; Van Haaften-Day C.; Hacker N. F.; De Bruijn H. W. A.; Van Der Zee A. G. J.; Jacobs I. J.; Fung E. T.; Chan D. W. Three Biomarkers Identified from Serum Proteomic Analysis for the Detection of Early Stage Ovarian Cancer. Cancer Res. 2004, 64, 5882–5890. 10.1158/0008-5472.CAN-04-0746. 15313933

[ref33] Galle P. R.; Foerster F.; Kudo M.; Chan S. L.; Llovet J. M.; Qin S.; Schelman W. R.; Chintharlapalli S.; Abada P. B.; Sherman M.; Zhu A. X. Biology and Significance of Alpha-Fetoprotein in Hepatocellular Carcinoma. Liver Int. 2019, 39, 2214–2229. 10.1111/LIV.14223. 31436873

[ref34] Lempiäinen A.; Stenman U. H.; Blomqvist C.; Hotakainen K. Free β-Subunit of Human Chorionic Gonadotropin in Serum Is a Diagnostically Sensitive Marker of Seminomatous Testicular Cancer. Clin. Chem. 2008, 54, 1840–1843. 10.1373/CLINCHEM.2008.108548. 18787014

[ref35] Richardson L. C.; Dowling N.; Henley J. An Update on Cancer Deaths in the United States; US Department of Health and Human Services, Centers for Disease Control and Prevention, Division of Cancer Prevention and Control: Atlanta, GA, 2022.

[ref36] Batth T. S.; Tollenaere M. A. X.; Rüther P.; Gonzalez-Franquesa A.; Prabhakar B. S.; Bekker-Jensen S.; Deshmukh A. S.; Olsen J. V. Protein Aggregation Capture on Microparticles Enables Multipurpose Proteomics Sample Preparation. Mol. Cell. Proteomics 2019, 18, 1027–1035. 10.1074/mcp.TIR118.001270. 30833379 PMC6495262

[ref37] Wiśniewski J. R.; Zougman A.; Nagaraj N.; Mann M. Universal Sample Preparation Method for Proteome Analysis. Nat. Methods 2009, 6, 359–362. 10.1038/nmeth.1322. 19377485

[ref38] Bruderer R.; Bernhardt O. M.; Gandhi T.; Xuan Y.; Sondermann J.; Schmidt M.; Gomez-Varela D.; Reiter L. Optimization of Experimental Parameters in Data-Independent Mass Spectrometry Significantly Increases Depth and Reproducibility of Results. Mol. Cell. Proteomics 2017, 16, 2296–2309. 10.1074/mcp.RA117.000314. 29070702 PMC5724188

[ref39] Bruderer R.; Bernhardt O. M.; Gandhi T.; Miladinović S. M.; Cheng L. Y.; Messner S.; Ehrenberger T.; Zanotelli V.; Butscheid Y.; Escher C.; Vitek O.; Rinner O.; Reiter L. Extending the Limits of Quantitative Proteome Profiling with Data-Independent Acquisition and Application to Acetaminophen-Treated Three-Dimensional Liver Microtissues. Mol. Cell. Proteomics 2015, 14, 1400–1410. 10.1074/mcp.M114.044305. 25724911 PMC4424408

[ref40] Kelstrup C. D.; Young C.; Lavallee R.; Nielsen M. L.; Olsen J. V. Optimized Fast and Sensitive Acquisition Methods for Shotgun Proteomics on a Quadrupole Orbitrap Mass Spectrometer. J. Proteome Res. 2012, 11, 3487–3497. 10.1021/pr3000249. 22537090

[ref41] Muntel J.; Gandhi T.; Verbeke L.; Bernhardt O. M.; Treiber T.; Bruderer R.; Reiter L. Surpassing 10 000 Identified and Quantified Proteins in a Single Run by Optimizing Current LC-MS Instrumentation and Data Analysis Strategy. Mol. Omi. 2019, 15, 348–360. 10.1039/C9MO00082H. 31465043

[ref42] Karpievitch Y. V.; Dabney A. R.; Smith R. D. Normalization and Missing Value Imputation for Label-Free LC-MS Analysis. BMC Bioinf. 2012, 13, S5 10.1186/1471-2105-13-S16-S5. PMC3489534 23176322

[ref43] Tsou C. C.; Avtonomov D.; Larsen B.; Tucholska M.; Choi H.; Gingras A. C.; Nesvizhskii A. I. DIA-Umpire: Comprehensive Computational Framework for Data-Independent Acquisition Proteomics. Nat. Methods 2015, 12, 258–264. 10.1038/nmeth.3255. 25599550 PMC4399776

[ref44] Ma Z.-Q.; Dasari S.; Chambers M. C.; Litton M. D.; Sobecki S. M.; Zimmerman L. J.; Halvey P. J.; Schilling B.; Drake P. M.; Gibson B. W.; Tabb D. L. IDPicker 2.0: Improved Protein Assembly with High Discrimination Peptide Identification Filtering. J. Proteome Res. 2009, 8, 3872 10.1021/PR900360J. 19522537 PMC2810655

[ref45] Lê Cao K.-A.; Boitard S.; Besse P. Sparse PLS Discriminant Analysis: Biologically Relevant Feature Selection and Graphical Displays for Multiclass Problems. BMC Bioinf. 2011, 12, 253 10.1186/1471-2105-12-253. PMC3133555 21693065

[ref46] Rohart F.; Gautier B.; Singh A.; Cao K.-A. L. MixOmics: An R Package for ‘omics Feature Selection and Multiple Data Integration. PLOS Comput. Biol. 2017, 13, e1005752 10.1371/JOURNAL.PCBI.1005752. 29099853 PMC5687754

[ref47] Eden E.; Navon R.; Steinfeld I.; Lipson D.; Yakhini Z. GOrilla: A Tool for Discovery and Visualization of Enriched GO Terms in Ranked Gene Lists. BMC Bioinf. 2009, 10, 48 10.1186/1471-2105-10-48. PMC2644678 19192299

[ref48] Dayon L.; Núñez Galindo A.; Cominetti O.; Corthésy J.; Kussmann M. A Highly Automated Shotgun Proteomic Workflow: Clinical Scale and Robustness for Biomarker Discovery in Blood. In Serum/Plasma Proteomics: Methods and Protocols, Greening D. W.; Simpson R. J., Eds.; Springer Science+Business Media, 2017; Vol. 1619, pp 433–449. 10.1007/978-1-4939-7057-5_30 28674902

[ref49] Wishart D. S.; Feunang Y. D.; Guo A. C.; Lo E. J.; Marcu A.; Grant J. R.; Sajed T.; Johnson D.; Li C.; Sayeeda Z.; Assempour N.; Iynkkaran I.; Liu Y.; MacIejewski A.; Gale N.; Wilson A.; Chin L.; Cummings R.; Le Di.; Pon A.; Knox C.; Wilson M. DrugBank 5.0: A Major Update to the DrugBank Database for 2018. Nucleic Acids Res. 2018, 46, D1074–D1082. 10.1093/NAR/GKX1037. 29126136 PMC5753335

[ref50] Pernemalm M.; Sandberg A.; Zhu Y.; Boekel J.; Tamburro D.; Schwenk J. M.; Björk A.; Wahren-Herlenius M.; Åmark H.; Östenson C.-G.; Westgren M.; Lehtiö J. In-Depth Human Plasma Proteome Analysis Captures Tissue Proteins and Transfer of Protein Variants across the Placenta. eLife 2019, 8, e41608 10.7554/eLife.41608. 30958262 PMC6519984

[ref51] Boerwinkle E.; Menzel H. J.; Kraft H. G.; Utermann G. Genetics of the Quantitative Lp(a) Lipoprotein Trait. III. Contribution of Lp(a) Glycoprotein Phenotypes to Normal Lipid Variation. Hum. Genet. 1989, 82, 73–78. 10.1007/BF00288277. 2523852

[ref52] Utermann G. The Mysteries of Lipoprotein(A). Science 1989, 246, 904–910. 10.1126/SCIENCE.2530631. 2530631

[ref53] Guo R. F.; Ward P. A. Role of C5a in Inflammatory Responses. Annu. Rev. Immunol. 2005, 23, 821–852. 10.1146/ANNUREV.IMMUNOL.23.021704.115835. 15771587

[ref54] Campbell P. J.; Getz G.; Korbel J. O.; Stuart J. M.; Jennings J. L.; Stein L. D.; Perry M. D.; Nahal-Bose H. K.; Ouellette B. F. F.; Li C. H.; Rheinbay E.; Nielsen G. P.; Sgroi D. C.; Wu C. L.; Faquin W. C.; Deshpande V.; Boutros P. C.; Lazar A. J.; Hoadley K. A.; Louis D. N.; Dursi L. J.; Yung C. K.; Bailey M. H.; Saksena G.; Raine K. M.; Buchhalter I.; Kleinheinz K.; Schlesner M.; Zhang J.; Wang W.; Wheeler D. A.; Ding L.; Simpson J. T.; O’Connor B. D.; Yakneen S.; Ellrott K.; Miyoshi N.; Butler A. P.; Royo R.; Shorser S. I.; Vazquez M.; Rausch T.; Tiao G.; Waszak S. M.; Rodriguez-Martin B.; Shringarpure S.; Wu D. Y.; Demidov G. M.; Delaneau O.; Hayashi S.; Imoto S.; Habermann N.; Segre A. V.; Garrison E.; Cafferkey A.; Alvarez E. G.; Heredia-Genestar J. M.; Muyas F.; Drechsel O.; Bruzos A. L.; Temes J.; Zamora J.; Baez-Ortega A.; Kim H. L.; Mashl R. J.; Ye K.; DiBiase A.; Huang K. lin.; Letunic I.; McLellan M. D.; Newhouse S. J.; Shmaya T.; Kumar S.; Wedge D. C.; Wright M. H.; Yellapantula V. D.; Gerstein M.; Khurana E.; Marques-Bonet T.; Navarro A.; Bustamante C. D.; Siebert R.; Nakagawa H.; Easton D. F.; Ossowski S.; Tubio J. M. C.; De La Vega F. M.; Estivill X.; Yuen D.; Mihaiescu G. L.; Omberg L.; Ferretti V.; Sabarinathan R.; Pich O.; Gonzalez-Perez A.; Taylor-Weiner A.; Fittall M. W.; Demeulemeester J.; Tarabichi M.; Roberts N. D.; Van Loo P.; Cortés-Ciriano I.; Urban L.; Park P.; Zhu B.; Pitkänen E.; Li Y.; Saini N.; Klimczak L. J.; Weischenfeldt J.; Sidiropoulos N.; Alexandrov L. B.; Rabionet R.; Escaramis G.; Bosio M.; Holik A. Z.; Susak H.; Prasad A.; Erkek S.; Calabrese C.; Raeder B.; Harrington E.; Mayes S.; Turner D.; Juul S.; Roberts S. A.; Song L.; Koster R.; Mirabello L.; Hua X.; Tanskanen T. J.; Tojo M.; Chen J.; Aaltonen L. A.; Rätsch G.; Schwarz R. F.; Butte A. J.; Brazma A.; Chanock S. J.; Chatterjee N.; Stegle O.; Harismendy O.; Bova G. S.; Gordenin D. A.; Haan D.; Sieverling L.; Feuerbach L.; Chalmers D.; Joly Y.; Knoppers B. M.; Molnár-Gábor F.; Phillips M.; Thorogood A.; Townend D.; Goldman M.; Fonseca N. A.; Xiang Q.; Craft B.; Piñeiro-Yáñez E.; Muñoz A.; Petryszak R.; Füllgrabe A.; Al-Shahrour F.; Keays M.; Haussler D.; Weinstein J.; Huber W.; Valencia A.; Papatheodorou I.; Zhu J.; Fan Y.; Torrents D.; Bieg M.; Chen K.; Chong Z.; Cibulskis K.; Eils R.; Fulton R. S.; Gelpi J. L.; Gonzalez S.; Gut I. G.; Hach F.; Heinold M.; Hu T.; Huang V.; Hutter B.; Jäger N.; Jung J.; Kumar Y.; Lalansingh C.; Leshchiner I.; Livitz D.; Ma E. Z.; Maruvka Y. E.; Milovanovic A.; Nielsen M. M.; Paramasivam N.; Pedersen J. S.; Puiggròs M.; Sahinalp S. C.; Sarrafi I.; Stewart C.; Stobbe M. D.; Wala J. A.; Wang J.; Wendl M.; Werner J.; Wu Z.; Xue H.; Yamaguchi T. N.; Yellapantula V.; Davis-Dusenbery B. N.; Grossman R. L.; Kim Y.; Heinold M. C.; Hinton J.; Jones D. R.; Menzies A.; Stebbings L.; Hess J. M.; Rosenberg M.; Dunford A. J.; Gupta M.; Imielinski M.; Meyerson M.; Beroukhim R.; Reimand J.; Dhingra P.; Favero F.; Dentro S.; Wintersinger J.; Rudneva V.; Park J. W.; Hong E. P.; Heo S. G.; Kahles A.; Lehmann K. Van.; Soulette C. M.; Shiraishi Y.; Liu F.; He Y.; Demircioğlu D.; Davidson N. R.; Greger L.; Li S.; Liu D.; Stark S. G.; Zhang F.; Amin S. B.; Bailey P.; Chateigner A.; Frenkel-Morgenstern M.; Hou Y.; Huska M. R.; Kilpinen H.; Lamaze F. C.; Li C.; Li X.; Li X.; Liu X.; Marin M. G.; Markowski J.; Nandi T.; Ojesina A. I.; Pan-Hammarström Q.; Park P. J.; Pedamallu C. S.; Su H.; Tan P.; Teh B. T.; Wang J.; Xiong H.; Ye C.; Yung C.; Zhang X.; Zheng L.; Zhu S.; Awadalla P.; Creighton C. J.; Wu K.; Yang H.; Göke J.; Zhang Z.; Brooks A. N.; Fittall M. W.; Martincorena I.; Rubio-Perez C.; Juul M.; Schumacher S.; Shapira O.; Tamborero D.; Mularoni L.; Hornshøj H.; Deu-Pons J.; Muiños F.; Bertl J.; Guo Q.; Gonzalez-Perez A.; Xiang Q.; Bazant W.; Barrera E.; Al-Sedairy S. T.; Aretz A.; Bell C.; Betancourt M.; Buchholz C.; Calvo F.; Chomienne C.; Dunn M.; Edmonds S.; Green E.; Gupta S.; Hutter C. M.; Jegalian K.; Jones N.; Lu Y.; Nakagama H.; Nettekoven G.; Planko L.; Scott D.; Shibata T.; Shimizu K.; Stratton M. R.; Yugawa T.; Tortora G.; VijayRaghavan K.; Zenklusen J. C.; Townend D.; Aminou B.; Bartolome J.; Boroevich K. A.; Boyce R.; Buchanan A.; Byrne N. J.; Chen Z.; Cho S.; Choi W.; Clapham P.; Dow M. T.; Dursi L. J.; Eils J.; Farcas C.; Fayzullaev N.; Flicek P.; Heath A. P.; Hofmann O.; Hong J. H.; Hudson T. J.; Hübschmann D.; Ivkovic S.; Jeon S. H.; Jiao W.; Kabbe R.; Kahles A.; Kerssemakers J. N. A.; Kim H.; Kim J.; Koscher M.; Koures A.; Kovacevic M.; Lawerenz C.; Liu J.; Mijalkovic S.; Mijalkovic-Lazic A. M.; Miyano S.; Nastic M.; Nicholson J.; Ocana D.; Ohi K.; Ohno-Machado L.; Pihl T. D.; Prinz M.; Radovic P.; Short C.; Sofia H. J.; Spring J.; Struck A. J.; Tijanic N.; Vicente D.; Wang Z.; Williams A.; Woo Y.; Wright A. J.; Yang L.; Hamilton M. P.; Johnson T. A.; Kahraman A.; Kellis M.; Polak P.; Sallari R.; Sinnott-Armstrong N.; von Mering C.; Beltran S.; Gerhard D. S.; Gut M.; Trotta J. R.; Whalley J. P.; Niu B.; Espiritu S. M. G.; Gao S.; Huang Y.; Lalansingh C. M.; Teague J. W.; Wendl M. C.; Abascal F.; Bader G. D.; Bandopadhayay P.; Barenboim J.; Brunak S.; Carlevaro-Fita J.; Chakravarty D.; Chan C. W. Y.; Choi J. K.; Diamanti K.; Fink J. L.; Frigola J.; Gambacorti-Passerini C.; Garsed D. W.; Haradhvala N. J.; Harmanci A. O.; Helmy M.; Herrmann C.; Hobolth A.; Hodzic E.; Hong C.; Isaev K.; Izarzugaza J. M. G.; Johnson R.; Juul R. I.; Kim J.; Kim J. K.; Jan Komorowski.; Lanzós A.; Larsson E.; Lee D.; Li S.; Li X.; Lin Z.; Liu E. M.; Lochovsky L.; Lou S.; Madsen T.; Marchal K.; Martinez-Fundichely A.; McGillivray P. D.; Meyerson W.; Paczkowska M.; Park K.; Park K.; Pons T.; Pulido-Tamayo S.; Reyes-Salazar I.; Reyna M. A.; Rubin M. A.; Salichos L.; Sander C.; Schumacher S. E.; Shackleton M.; Shen C.; Shrestha R.; Shuai S.; Tsunoda T.; Umer H. M.; Uusküla-Reimand L.; Verbeke L. P. C.; Wadelius C.; Wadi L.; Warrell J.; Wu G.; Yu J.; Zhang J.; Zhang X.; Zhang Y.; Zhao Z.; Zou L.; Lawrence M. S.; Raphael B. J.; Bailey P. J.; Craft D.; Goldman M. J.; Aburatani H.; Binder H.; Dinh H. Q.; Heath S. C.; Hoffmann S.; Imbusch C. D.; Kretzmer H.; Laird P. W.; Martin-Subero J. I.; Nagae G.; Shen H.; Wang Q.; Weichenhan D.; Zhou W.; Berman B. P.; Brors B.; Plass C.; Akdemir K. C.; Bowtell D. D. L.; Burns K. H.; Busanovich J.; Chan K.; Dueso-Barroso A.; Edwards P. A.; Etemadmoghadam D.; Haber J. E.; Jones D. T. W.; Ju Y. S.; Kazanov M. D.; Koh Y.; Kumar K.; Lee E. A.; Lee J. J. K.; Lynch A. G.; Macintyre G.; Markowetz F.; Navarro F. C. P.; Pearson J. V.; Rippe K.; Scully R.; Villasante I.; Waddell N.; Yang L.; Yao X.; Yoon S. S.; Zhang C. Z.; Bergstrom E. N.; Boot A.; Covington K.; Fujimoto A.; Huang M. N.; Islam S. M. A.; McPherson J. R.; Morganella S.; Mustonen V.; Ng A. W. T.; Prokopec S. D.; Vázquez-García I.; Wu Y.; Yousif F.; Yu W.; Rozen S. G.; Rudneva V. A.; Shringarpure S. S.; Turner D. J.; Xia T.; Atwal G.; Chang D. K.; Cooke S. L.; Faltas B. M.; Haider S.; Kaiser V. B.; Karlić R.; Kato M.; Kübler K.; Margolin A.; Martin S.; Nik-Zainal S.; P’ng C.; Semple C. A.; Smith J.; Sun R. X.; Thai K.; Wright D. W.; Yuan K.; Biankin A. V.; Garraway L.; Grimmond S. M.; Adams D. J.; Anur P.; Cao S.; Christie E. L.; Cmero M.; Cun Y.; Dawson K. J.; Dentro S. C.; Deshwar A. G.; Donmez N.; Drews R. M.; Gerstung M.; Ha G.; Haase K.; Jerman L.; Ji Y.; Jolly C.; Lee J.; Lee-Six H.; Malikic S.; Mitchell T. J.; Morris Q. D.; Oesper L.; Peifer M.; Peto M.; Rosebrock D.; Rubanova Y.; Salcedo A.; Sengupta S.; Shi R.; Shin S. J.; Spiro O.; Vembu S.; Wintersinger J. A.; Yang T. P.; Yu K.; Zhu H.; Spellman P. T.; Weinstein J. N.; Chen Y.; Fujita M.; Han L.; Hasegawa T.; Komura M.; Li J.; Mizuno S.; Shimizu E.; Wang Y.; Xu Y.; Yamaguchi R.; Yang F.; Yang Y.; Yoon C. J.; Yuan Y.; Liang H.; Alawi M.; Borozan I.; Brewer D. S.; Cooper C. S.; Desai N.; Grundhoff A.; Iskar M.; Su X.; Zapatka M.; Lichter P.; Alsop K.; Bruxner T. J. C.; Christ A. N.; Cordner S. M.; Cowin P. A.; Drapkin R.; Fereday S.; George J.; Hamilton A.; Holmes O.; Hung J. A.; Kassahn K. S.; Kazakoff S. H.; Kennedy C. J.; Leonard C. R.; Mileshkin L.; Miller D. K.; Arnau G. M.; Mitchell C.; Newell F.; Nones K.; Patch A. M.; Quinn M. C.; Taylor D. F.; Thorne H.; Traficante N.; Vedururu R.; Waddell N. M.; Waring P. M.; Wood S.; Xu Q.; deFazio A.; Anderson M. J.; Antonello D.; Barbour A. P.; Bassi C.; Bersani S.; Cataldo I.; Chantrill L. A.; Chiew Y. E.; Chou A.; Cingarlini S.; Cloonan N.; Corbo V.; Davi M. V.; Duthie F. R.; Gill A. J.; Graham J. S.; Harliwong I.; Jamieson N. B.; Johns A. L.; Kench J. G.; Landoni L.; Lawlor R. T.; Mafficini A.; Merrett N. D.; Miotto M.; Musgrove E. A.; Nagrial A. M.; Oien K. A.; Pajic M.; Pinese M.; Robertson A. J.; Rooman I.; Rusev B. C.; Samra J. S.; Scardoni M.; Scarlett C. J.; Scarpa A.; Sereni E.; Sikora K. O.; Simbolo M.; Taschuk M. L.; Toon C. W.; Vicentini C.; Wu J.; Zeps N.; Behren A.; Burke H.; Cebon J.; Dagg R. A.; De Paoli-Iseppi R.; Dutton-Regester K.; Field M. A.; Fitzgerald A.; Hersey P.; Jakrot V.; Johansson P. A.; Kakavand H.; Kefford R. F.; Lau L. M. S.; Long G. V.; Pickett H. A.; Pritchard A. L.; Pupo G. M.; Saw R. P. M.; Schramm S. J.; Shang C. A.; Shang P.; Spillane A. J.; Stretch J. R.; Tembe V.; Thompson J. F.; Vilain R. E.; Wilmott J. S.; Yang J. Y.; Hayward N. K.; Mann G. J.; Scolyer R. A.; Bartlett J.; Bavi P.; Chadwick D. E.; Chan-Seng-Yue M.; Cleary S.; Connor A. A.; Czajka K.; Denroche R. E.; Dhani N. C.; Eagles J.; Gallinger S.; Grant R. C.; Hedley D.; Hollingsworth M. A.; Jang G. H.; Johns J.; Kalimuthu S.; Liang S. Ben.; Lungu I.; Luo X.; Mbabaali F.; McPherson T. A.; Miller J. K.; Moore M. J.; Notta F.; Pasternack D.; Petersen G. M.; Roehrl M. H. A.; Sam M.; Selander I.; Serra S.; Shahabi S.; Thayer S. P.; Timms L. E.; Wilson G. W.; Wilson J. M.; Wouters B. G.; McPherson J. D.; Beck T. A.; Bhandari V.; Collins C. C.; Fleshner N. E.; Fox N. S.; Fraser M.; Heisler L. E.; Lalonde E.; Livingstone J.; Meng A.; Sabelnykova V. Y.; Shiah Y. J.; Van der Kwast T.; Bristow R. G.; Ding S.; Fan D.; Li L.; Nie Y.; Xiao X.; Xing R.; Yang S.; Yu Y.; Zhou Y.; Banks R. E.; Bourque G.; Brennan P.; Letourneau L.; Riazalhosseini Y.; Scelo G.; Vasudev N.; Viksna J.; Lathrop M.; Tost J.; Ahn S. M.; Aparicio S.; Arnould L.; Aure M. R.; Bhosle S. G.; Birney E.; Borg A.; Boyault S.; Brinkman A. B.; Brock J. E.; Broeks A.; Børresen-Dale A. L.; Caldas C.; Chin S. F.; Davies H.; Desmedt C.; Dirix L.; Dronov S.; Ehinger A.; Eyfjord J. E.; Fatima A.; Foekens J. A.; Futreal P. A.; Garred Ø.; Giri D. D.; Glodzik D.; Grabau D.; Hilmarsdottir H.; Hooijer G. K.; Jacquemier J.; Jang S. J.; Jonasson J. G.; Jonkers J.; Kim H. Y.; King T. A.; Knappskog S.; Kong G.; Krishnamurthy S.; Lakhani S. R.; Langerød A.; Larsimont D.; Lee H. J.; Lee J. Y.; Lee M. T. M.; Lingjærde O. C.; MacGrogan G.; Martens J. W. M.; O’Meara S.; Pauporté I.; Pinder S.; Pivot X.; Provenzano E.; Purdie C. A.; Ramakrishna M.; Ramakrishnan K.; Reis-Filho J.; Richardson A. L.; Ringnér M.; Rodriguez J. B.; Rodríguez-González F. G.; Romieu G.; Salgado R.; Sauer T.; Shepherd R.; Sieuwerts A. M.; Simpson P. T.; Smid M.; Sotiriou C.; Span P. N.; Stefánsson ÓA.; Stenhouse A.; Stunnenberg H. G.; Sweep F.; Tan B. K. T.; Thomas G.; Thompson A. M.; Tommasi S.; Treilleux I.; Tutt A.; Ueno N. T.; Van Laere S.; Van den Eynden G. G.; Vermeulen P.; Viari A.; Vincent-Salomon A.; Wong B. H.; Yates L.; Zou X.; van Deurzen C. H. M.; van de Vijver M. J.; van’t Veer L.; Ammerpohl O.; Aukema S.; Bergmann A. K.; Bernhart S. H.; Borkhardt A.; Borst C.; Burkhardt B.; Claviez A.; Goebler M. E.; Haake A.; Haas S.; Hansmann M.; Hoell J. I.; Hummel M.; Karsch D.; Klapper W.; Kneba M.; Kreuz M.; Kube D.; Küppers R.; Lenze D.; Loeffler M.; López C.; Mantovani-Löffler L.; Möller P.; Ott G.; Radlwimmer B.; Richter J.; Rohde M.; Rosenstiel P. C.; Rosenwald A.; Schilhabel M. B.; Schreiber S.; Stadler P. F.; Staib P.; Stilgenbauer S.; Sungalee S.; Szczepanowski M.; Toprak U. H.; Trümper L. H. P.; Wagener R.; Zenz T.; Hovestadt V.; von Kalle C.; Kool M.; Korshunov A.; Landgraf P.; Lehrach H.; Northcott P. A.; Pfister S. M.; Reifenberger G.; Warnatz H. J.; Wolf S.; Yaspo M. L.; Assenov Y.; Gerhauser C.; Minner S.; Schlomm T.; Simon R.; Sauter G.; Sültmann H.; Biswas N. K.; Maitra A.; Majumder P. P.; Sarin R.; Barbi S.; Bonizzato G.; Cantù C.; Dei Tos A. P.; Fassan M.; Grimaldi S.; Luchini C.; Malleo G.; Marchegiani G.; Milella M.; Paiella S.; Pea A.; Pederzoli P.; Ruzzenente A.; Salvia R.; Sperandio N.; Arai Y.; Hama N.; Hiraoka N.; Hosoda F.; Nakamura H.; Ojima H.; Okusaka T.; Totoki Y.; Urushidate T.; Fukayama M.; Ishikawa S.; Katai H.; Katoh H.; Komura D.; Rokutan H.; Saito-Adachi M.; Suzuki A.; Taniguchi H.; Tatsuno K.; Ushiku T.; Yachida S.; Yamamoto S.; Aikata H.; Arihiro K.; Ariizumi S. ichi.; Chayama K.; Furuta M.; Gotoh K.; Hayami S.; Hirano S.; Kawakami Y.; Maejima K.; Nakamura T.; Nakano K.; Ohdan H.; Sasaki-Oku A.; Tanaka H.; Ueno M.; Yamamoto M.; Yamaue H.; Choo S. P.; Cutcutache I.; Khuntikeo N.; Ong C. K.; Pairojkul C.; Popescu I.; Ahn K. S.; Aymerich M.; Lopez-Guillermo A.; López-Otín C.; Puente X. S.; Campo E.; Amary F.; Baumhoer D.; Behjati S.; Bjerkehagen B.; Futreal P. A.; Myklebost O.; Pillay N.; Tarpey P.; Tirabosco R.; Zaikova O.; Flanagan A. M.; Boultwood J.; Bowen D. T.; Cazzola M.; Green A. R.; Hellstrom-Lindberg E.; Malcovati L.; Nangalia J.; Papaemmanuil E.; Vyas P.; Ang Y.; Barr H.; Beardsmore D.; Eldridge M.; Gossage J.; Grehan N.; Hanna G. B.; Hayes S. J.; Hupp T. R.; Khoo D.; Lagergren J.; Lovat L. B.; MacRae S.; O’Donovan M.; O’Neill J. R.; Parsons S. L.; Preston S. R.; Puig S.; Roques T.; Sanders G.; Sothi S.; Tavaré S.; Tucker O.; Turkington R.; Underwood T. J.; Welch I.; Fitzgerald R. C.; Berney D. M.; De Bono J. S.; Cahill D.; Camacho N.; Dennis N. M.; Dudderidge T.; Edwards S. E.; Fisher C.; Foster C. S.; Ghori M.; Gill P.; Gnanapragasam V. J.; Gundem G.; Hamdy F. C.; Hawkins S.; Hazell S.; Howat W.; Isaacs W. B.; Karaszi K.; Kay J. D.; Khoo V.; Kote-Jarai Z.; Kremeyer B.; Kumar P.; Lambert A.; Leongamornlert D. A.; Livni N.; Lu Y. J.; Luxton H. J.; Marsden L.; Massie C. E.; Matthews L.; Mayer E.; McDermott U.; Merson S.; Neal D. E.; Ng A.; Nicol D.; Ogden C.; Rowe E. W.; Shah N. C.; Thomas S.; Thompson A.; Verrill C.; Visakorpi T.; Warren A. Y.; Whitaker H. C.; Zhang H.; van As N.; Eeles R. A.; Abeshouse A.; Agrawal N.; Akbani R.; Al-Ahmadie H.; Albert M.; Aldape K.; Ally A.; Appelbaum E. L.; Armenia J.; Asa S.; Auman J. T.; Balasundaram M.; Balu S.; Barnholtz-Sloan J.; Bathe O. F.; Baylin S. B.; Benz C.; Berchuck A.; Berrios M.; Bigner D.; Birrer M.; Bodenheimer T.; Boice L.; Bootwalla M. S.; Bosenberg M.; Bowlby R.; Boyd J.; Broaddus R. R.; Brock M.; Brooks D.; Bullman S.; Caesar-Johnson S. J.; Carey T. E.; Carlsen R.; Cerfolio R.; Chandan V. S.; Chen H. W.; Cherniack A. D.; Chien J.; Cho J.; Chuah E.; Cibulskis C.; Cope L.; Cordes M. G.; Curley E.; Czerniak B.; Danilova L.; Davis I. J.; Defreitas T.; Demchok J. A.; Dhalla N.; Dhir R.; Doddapaneni H. V.; El-Naggar A.; Felau I.; Ferguson M. L.; Finocchiaro G.; Fong K. M.; Frazer S.; Friedman W.; Fronick C. C.; Fulton L. A.; Gabriel S. B.; Gao J.; Gehlenborg N.; Gershenwald J. E.; Ghossein R.; Giama N. H.; Gibbs R. A.; Gomez C.; Govindan R.; Hayes D. N.; Hegde A. M.; Heiman D. I.; Heins Z.; Hepperla A. J.; Holbrook A.; Holt R. A.; Hoyle A. P.; Hruban R. H.; Hu J.; Huang M.; Huntsman D.; Huse J.; Iacobuzio-Donahue C. A.; Ittmann M.; Jayaseelan J. C.; Jefferys S. R.; Jones C. D.; Jones S. J. M.; Juhl H.; Kang K. J.; Karlan B.; Kasaian K.; Kebebew E.; Kim H. K.; Korchina V.; Kundra R.; Lai P. H.; Lander E.; Le X.; Lee D.; Levine D. A.; Lewis L.; Ley T.; Li H. I.; Lin P.; Linehan W. M.; Liu F. F.; Lu Y.; Lype L.; Ma Y.; Maglinte D. T.; Mardis E. R.; Marks J.; Marra M. A.; Matthew T. J.; Mayo M.; McCune K.; Meier S. R.; Meng S.; Mieczkowski P. A.; Mikkelsen T.; Miller C. A.; Mills G. B.; Moore R. A.; Morrison C.; Mose L. E.; Moser C. D.; Mungall A. J.; Mungall K.; Mutch D.; Muzny D. M.; Myers J.; Newton Y.; Noble M. S.; O’Donnell P.; O’Neill B. P.; Ochoa A.; Park J. W.; Parker J. S.; Pass H.; Pastore A.; Pennell N. A.; Perou C. M.; Petrelli N.; Potapova O.; Rader J. S.; Ramalingam S.; Rathmell W. K.; Reuter V.; Reynolds S. M.; Ringel M.; Roach J.; Roberts L. R.; Robertson A. G.; Sadeghi S.; Saller C.; Sanchez-Vega F.; Schadendorf D.; Schein J. E.; Schmidt H. K.; Schultz N.; Seethala R.; Senbabaoglu Y.; Shelton T.; Shi Y.; Shih J.; Shmulevich I.; Shriver C.; Signoretti S.; Simons J. V.; Singer S.; Sipahimalani P.; Skelly T. J.; Smith-McCune K.; Socci N. D.; Soloway M. G.; Sood A. K.; Tam A.; Tan D.; Tarnuzzer R.; Thiessen N.; Thompson R. H.; Thorne L. B.; Tsao M.; Umbricht C.; Van Den Berg D. J.; Van Meir E. G.; Veluvolu U.; Voet D.; Wang L.; Weinberger P.; Weisenberger D. J.; Wigle D.; Wilkerson M. D.; Wilson R. K.; Winterhoff B.; Wiznerowicz M.; Wong T.; Wong W.; Xi L.; Yau C.; Zhang H.; Zhang H.; Zhang J. Pan-Cancer Analysis of Whole Genomes. Nature 2020, 578, 82–93. 10.1038/s41586-020-1969-6. 32025007 PMC7025898

[ref55] Powell D. J.; De Vries C. R.; Allen T.; Ahmadzadeh M.; Rosenberg S. A. Inability to Mediate Prolonged Reduction of Regulatory T Cells After Transfer of Autologous CD25-Depleted PBMC and Interleukin-2 After Lymphodepleting Chemotherapy. J. Immunother. 2007, 30, 438 10.1097/CJI.0B013E3180600FF9. 17457218 PMC2140222

[ref56] Afzal N.; Javaid K.; Zaman S.; Zafar A.; Nagi A. H. Enumeration of CD4(+)CD25(+)T Regulatory Cells in Type-II Diabetes Retinopathy. Pak. J. Pharm. Sci. 2014, 27, 1191–1197. 25176359

[ref57] Liot S.; Aubert A.; Hervieu V.; Kholti N.; El; Schalkwijk J.; Verrier B.; Valcourt U.; Lambert E. Loss of Tenascin-X Expression during Tumor Progression: A New Pan-Cancer Marker. Matrix Biol. Plus 2020, 6–7, 100021 10.1016/J.MBPLUS.2020.100021. PMC7852205 33543019

[ref58] Lakkim V.; Reddy M. C.; Prasad D. V. R.; Lomada D. Role of STAT3 in Colorectal Cancer Development. Role Transcr. Factors Gastrointest. Malig. 2017, 269–298. 10.1007/978-981-10-6728-0_19.

[ref59] Zhou H. M.; Fang Y. Y.; Weinberger P. M.; Ding L. L.; Cowell J. K.; Hudson F. Z.; Ren M.; Lee J. R.; Chen Q. K.; Su H.; Dynan W. S.; Lin Y. Transgelin Increases Metastatic Potential of Colorectal Cancer Cells in Vivo and Alters Expression of Genes Involved in Cell Motility. BMC Cancer 2016, 16, 55 10.1186/s12885-016-2105-8. 26847345 PMC4741053

[ref60] Hu T.; Liu H.; Liang Z.; Wang F.; Zhou C.; Zheng X.; Zhang Y.; Song Y.; Hu J.; He X.; Xiao J.; King R. J.; Wu X.; Lan P. Tumor-Intrinsic CD47 Signal Regulates Glycolysis and Promotes Colorectal Cancer Cell Growth and Metastasis. Theranostics 2020, 10, 4056 10.7150/THNO.40860. 32226539 PMC7086360

[ref61] Takadate T.; Onogawa T.; Fukuda T.; Motoi F.; Suzuki T.; Fujii K.; Kihara M.; Mikami S.; Bando Y.; Maeda S.; Ishida K.; Minowa T.; Hanagata N.; Ohtsuka H.; Katayose Y.; Egawa S.; Nishimura T.; Unno M. Novel Prognostic Protein Markers of Resectable Pancreatic Cancer Identified by Coupled Shotgun and Targeted Proteomics Using Formalin-Fixed Paraffin-Embedded Tissues. Int. J. Cancer 2013, 132, 1368–1382. 10.1002/IJC.27797. 22915188

[ref62] Grønborg M.; Kristiansen T. Z.; Iwahori A.; Chang R.; Reddy R.; Sato N.; Molina H.; Jensen O. N.; Hruban R. H.; Goggins M. G.; Maitra A.; Pandey A. Biomarker Discovery from Pancreatic Cancer Secretome Using a Differential Proteomic Approach. Mol. Cell. Proteomics 2006, 5, 157–171. 10.1074/MCP.M500178-MCP200. 16215274

[ref63] Turtoi A.; Musmeci D.; Wang Y.; Dumont B.; Somja J.; Bevilacqua G.; De Pauw E.; Delvenne P.; Castronovo V. Identification of Novel Accessible Proteins Bearing Diagnostic and Therapeutic Potential in Human Pancreatic Ductal Adenocarcinoma. J. Proteome Res. 2011, 10, 4302–4313. 10.1021/PR200527Z. 21755970

[ref64] Sinclair J.; Timms J. F. Quantitative Profiling of Serum Samples Using TMT Protein Labelling, Fractionation and LC-MS/MS. Methods 2011, 54, 361–369. 10.1016/J.YMETH.2011.03.004. 21397697

[ref65] Chen R.; Pan S.; Yi E. C.; Donohoe S.; Bronner M. P.; Potter J. D.; Goodlett D. R.; Aebersold R.; Brentnall T. A. Quantitative Proteomic Profiling of Pancreatic Cancer Juice. Proteomics 2006, 6, 3871–3879. 10.1002/PMIC.200500702. 16739137

[ref66] Wang V. M. Y.; Ferreira R. M. M.; Almagro J.; Evan T.; Legrave N.; Zaw Thin M.; Frith D.; Carvalho J.; Barry D. J.; Snijders A. P.; Herbert E.; Nye E. L.; MacRae J. I.; Behrens A. CD9 Identifies Pancreatic Cancer Stem Cells and Modulates Glutamine Metabolism to Fuel Tumour Growth. Nat. Cell Biol. 2019, 21, 1425–1435. 10.1038/s41556-019-0407-1. 31685994 PMC6944508

[ref67] Zhu J.; He J.; Liu Y.; Simeone D. M.; Lubman D. M. Identification of Glycoprotein Markers for Pancreatic Cancer CD24 +CD44 + Stem-like Cells Using Nano-LC-MS/MS and Tissue Microarray. J. Proteome Res. 2012, 11, 2272–2281. 10.1021/pr201059g. 22335271 PMC3321127

[ref68] Resovi A.; Borsotti P.; Ceruti T.; Passoni A.; Zucchetti M.; Berndt A.; Riser B. L.; Taraboletti G.; Belotti D. CCN-Based Therapeutic Peptides Modify Pancreatic Ductal Adenocarcinoma Microenvironment and Decrease Tumor Growth in Combination with Chemotherapy. Cells 2020, 9, 952 10.3390/cells9040952. PMC7226963 32294968

[ref69] Sharen G.; Peng Y.; Cheng H.; Liu Y.; Shi Y.; Zhao J. Prognostic Value of GLUT-1 Expression in Pancreatic Cancer: Results from 538 Patients. Oncotarget 2017, 8, 19760 10.18632/ONCOTARGET.15035. 28178665 PMC5386719

[ref70] Jayaraman A. K.; Jayaraman S. Increased Level of Exogenous Zinc Induces Cytotoxicity and Up-Regulates the Expression of the ZnT-1 Zinc Transporter Gene in Pancreatic Cancer Cells. J. Nutr. Biochem. 2011, 22, 79–88. 10.1016/J.JNUTBIO.2009.12.001. 20392624

[ref71] Topol E. J. High-Performance Medicine: The Convergence of Human and Artificial Intelligence. Nat. Med. 2019, 25, 44–56. 10.1038/s41591-018-0300-7. 30617339

[ref72] Goecks J.; Jalili V.; Heiser L. M.; Gray J. W. How Machine Learning Will Transform Biomedicine. Cell 2020, 181, 92–101. 10.1016/J.CELL.2020.03.022. 32243801 PMC7141410

[ref73] Choi M.; Carver J.; Chiva C.; Tzouros M.; Huang T.; Tsai T. H.; Pullman B.; Bernhardt O. M.; Hüttenhain R.; Teo G. C.; Perez-Riverol Y.; Muntel J.; Müller M.; Goetze S.; Pavlou M.; Verschueren E.; Wollscheid B.; Nesvizhskii A. I.; Reiter L.; Dunkley T.; Sabidó E.; Bandeira N.; Vitek O. MassIVE.Quant: A Community Resource of Quantitative Mass Spectrometry–Based Proteomics Datasets. Nat. Methods 2020, 17, 981–984. 10.1038/s41592-020-0955-0. 32929271 PMC7541731

