# Supplementary material for: Biomarker Candidates for Tumors Identified from Deep-Profiled Plasma Stem Predominantly from the Low Abundant Area
Source: J Proteome Res. 2022 May 23;21(7):1718–35. doi: 10.1021/acs.jproteome.2c00122 (PMC9251764; doi:10.1021/acs.jproteome.2c00122)
Supplement: Supplementary file 1 — pr2c00122_si_001.pdf [file pr2c00122_si_001.pdf]

Biomarker candidates for tumors identified from deep-profiled plasma stem predominantly from the low abundant area

Marco Tognetti[1,#], Kamil Sklodowski[1,#], Sebastian Müller[1], Dominique Kamber[1], Jan Muntel[1], Roland Bruderer[1,*] and Lukas Reiter[1,*]

[1]Biognosys, 8952 Schlieren, Zurich, Switzerland

[#]These authors contributed equally: Marco Tognetti, Kamil Sklodowski

[*]These authors jointly supervised this work: Lukas Reiter, Roland Bruderer. ✉email: lukas.reiter@biognosys.com; roland.bruderer@biognosys.com

Table of Content

Suppl. Fig. 1: Controlled quantitative experiment with plasma and mass spectrometric performance benchmarking. *S-2*

Suppl. Fig. 2: Deep plasma discovery proteomics of five solid cancer types. *S-3*

Suppl. Fig. 3: Pan-cancer predictive model based on deeply profiled plasma. *S-4*

Suppl. Fig. 4: Biomarker candidates within and across the five solid cancers. *S-5*

Suppl. Fig. 5: Colorectal cancer analysis. *S-6*

Suppl. Fig. 6: Pancreatic cancer analysis. *S-7*

Suppl. Fig. 7: Identifications for the cancer plasma study in dependence of gradient length. *S-8*

Suppl. Table 1: Sample origin table. *Supporting_Table_1.XLS*

Suppl. Table 2: Cancer cohort composition table. *Supporting_Table_2.XLS*

Suppl. Table 3: List of the differentially abundant proteins between healthy, early and late stage cancer using univariate analysis. *Supporting_Table_3.XLSX*

Suppl. Table 4: List of the proteins selected by sPLSDA for each cancer type along with their importance. *Supporting_Table_4.XLSX*

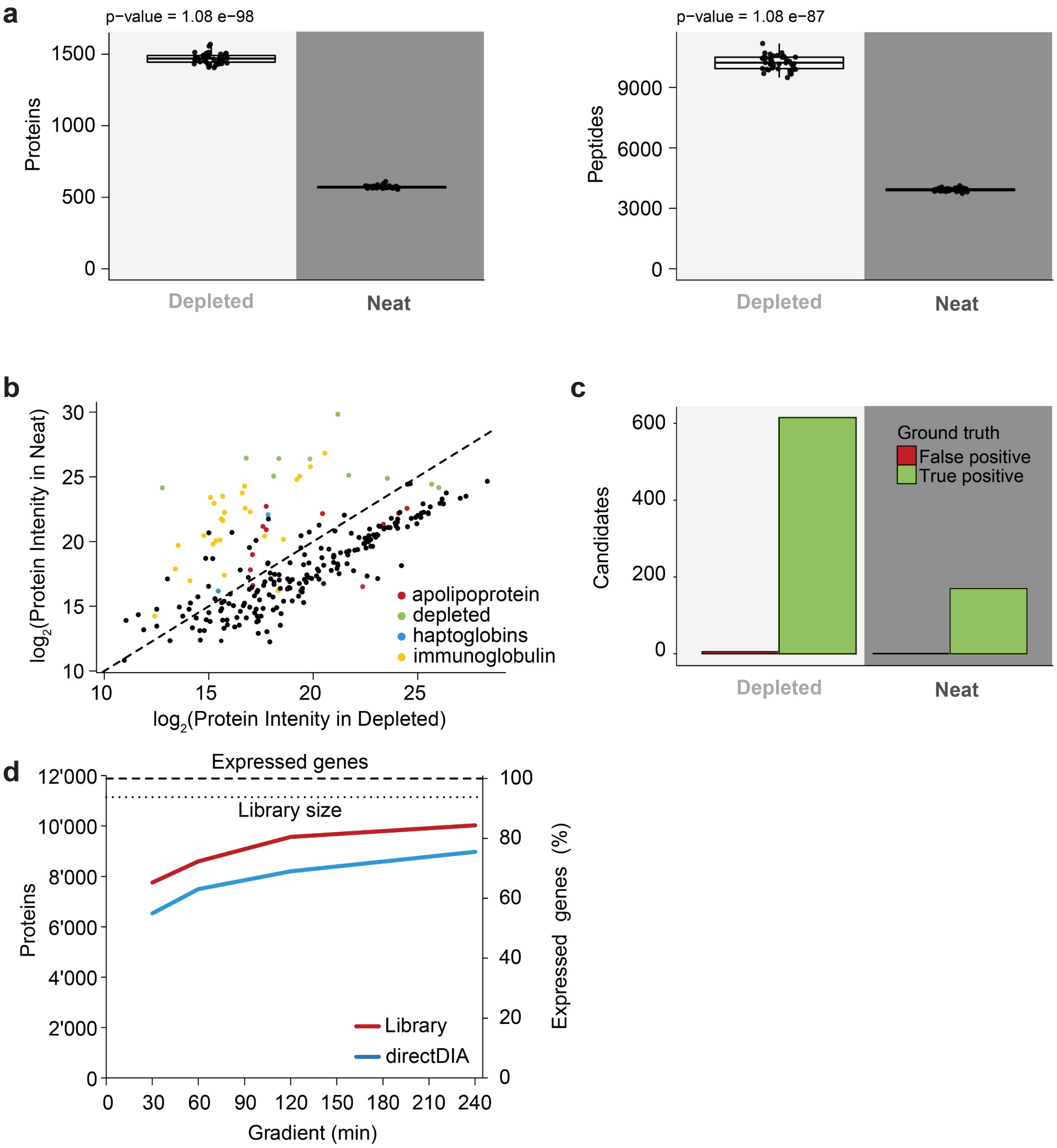

Suppl. Fig. 1: Controlled quantitative experiment with plasma and mass spectrometric performance benchmarking. (a) Boxplot visualization of the number of identified protein (protein groups) and peptides (stripped sequences) of the controlled quantitative experiment with neat and depleted plasma. Thick lines indicate medians, boxes indicate the 25% and 75% quantiles, whiskers extend between the median and ± (1.58 × inter-quantile range) and each data point represents a sample (n=80). T-test results are overlaid. **(b)** Correlation plot between log-transformed intensities in the neat (y-axis) and depleted (x-axis) datasets. The dash line represents perfect correlation. Points represent single proteins and are colored according to their relationship to the depleted proteins in their protein description. **(c)** Representation of the t-test candidates (FDR estimation by the Storey method) divided into true positives and false positives based on the ground truth for the controlled quantitative experiment of both the depleted and neat set. **(d)** Representation of the number of protein identifications from a Pierce-HeLa digest using the optimized FAIMS-DIA methods at increasing gradient lengths. The expressed genes number is taken from the human protein atlas and is represented by the thick dashed line (RNAseq data, https://www.proteinatlas.org). The thin dotted line represents the library size.

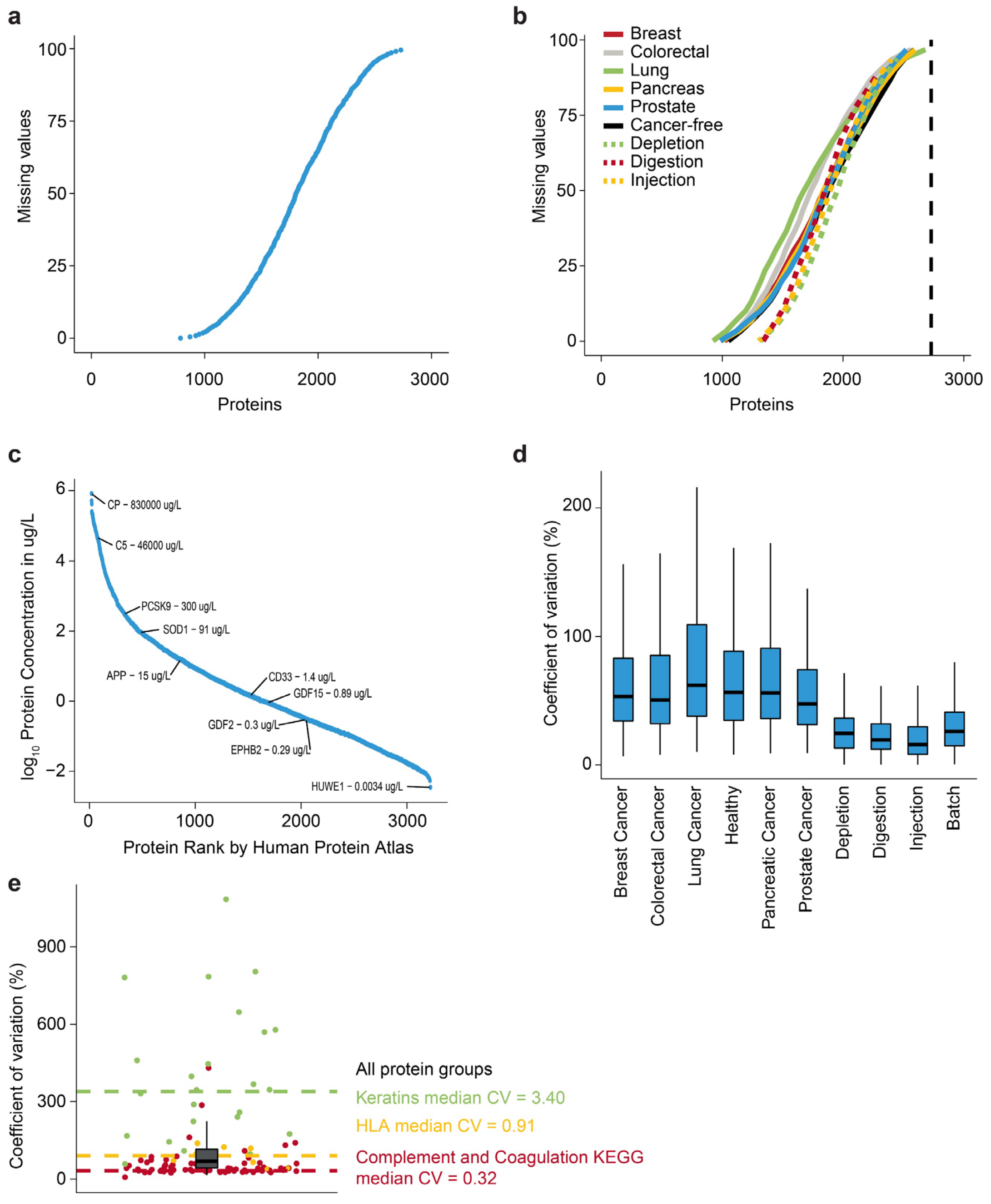

Suppl. Fig. 2: Deep plasma discovery proteomics of five solid cancer types. (a) Percentage of missing values in the cohort study plotted against the number of proteins (protein groups) with that value or less. **(b)** Same as in panel *a* but for different subsets in the study (n=30 for all but injection n=15, digestion n=9 and depletion n=16). The dash line represents the cumulatively identified proteins across the study. **(c)** Plot of protein rank by the Human Protein Atlas vs. log-transformed reported protein concentration of the identified proteins, spanning 8 orders of magnitude dynamic range as reported in the Human Protein Atlas (3,222 proteins detected in human plasma by mass spectrometry, of which 70% were identified and quantified in this work). Selected proteins were labeled along with the reported concentration. **(d)** Boxplot representation of the coefficient of variation (CV) of the quality control measurements across the processing steps and of the biological variance across cancer types. The CV was calculated on each level: injection (median CV=16%), digestion (CV=19%), depletion (CV=25%), and column (CV=26%). Thick lines indicate medians, boxes indicate the 25% and 75% quantiles, and whiskers extend between the median and ± (1.58 × inter-quantile range). **(e)** Boxplot representation of the biological coefficient of variation across all biological samples (n=180) as in panel *d*. Selected biological pathways are overlaid as points and dashed lines for individual proteins and the median, respectively.

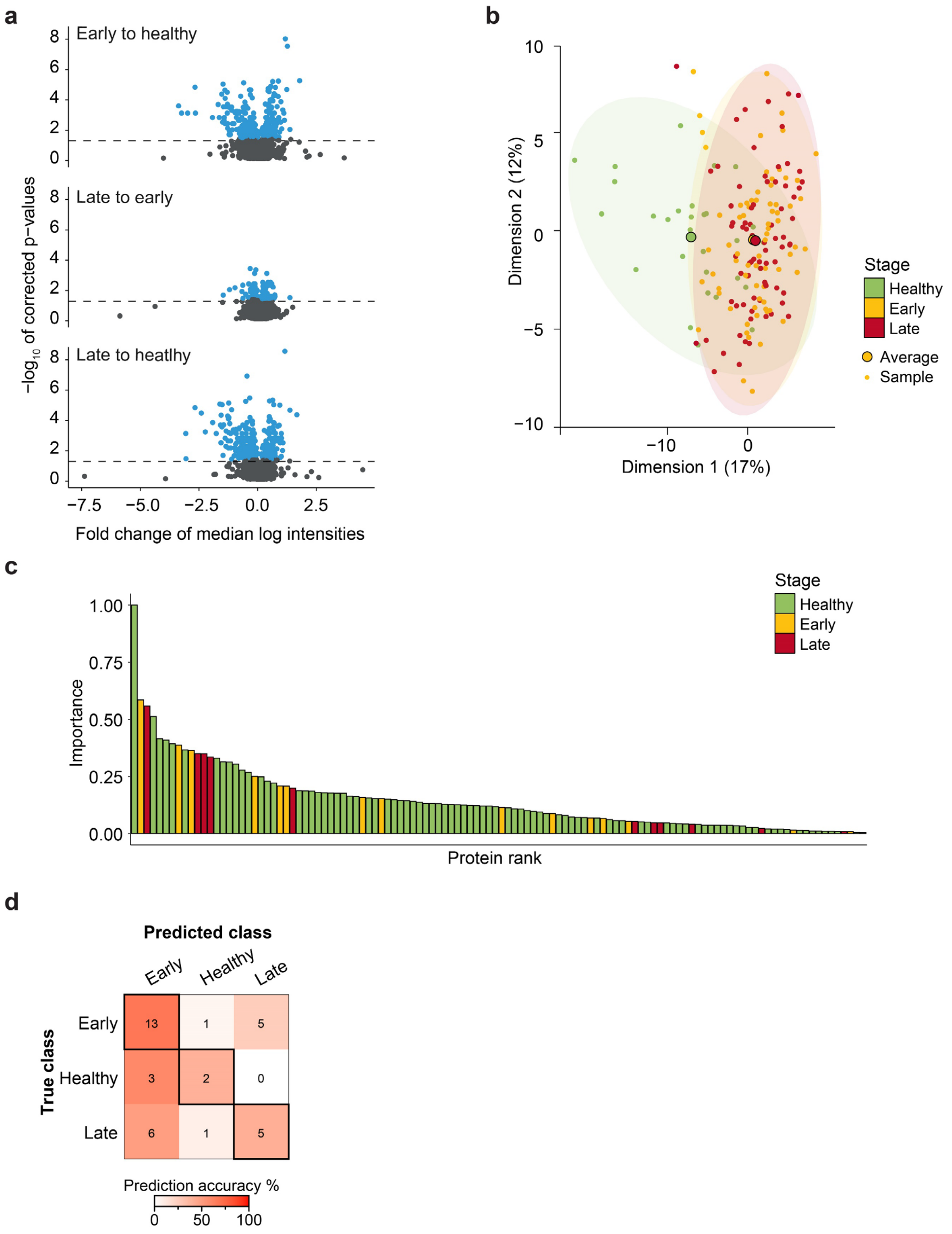

Suppl. Fig. 3: Pan-cancer predictive model based on deeply profiled plasma. (a) Log-transformed median fold change vs. $-\log_{10}$ p-value for all proteins for the three-way comparisons (heathy, early and late stage) using univariate comparison (Pairwise Wilcoxon Rank Sum Tests) for each protein with all 180 samples. The threshold for protein selection is represented as a dashed line at a p-value 0.05. Proteins with a within-group corrected p-value below 0.05 are depicted in blue. **(b)** Representation of the first two dimensions from the PCA analysis of sPLSDA identified candidates in pan-cancer analysis. Small points represent samples and large points the average across the stage (n=180). The first dimension separates healthy from diseased samples and explains 17% of the variance in the data. Corresponding ellipses represent sample concentration around the mean. **(c)** Representation of the sPLSDA selected biomarker candidates (94 in total) for the pan-cancer model ordered by relative importance and colored by the stage. **(d)** Overview of the classification accuracy of the machine learning model for the pan-cancer validation set (n=36). Correct classifications are represented in the highlighted boxes.

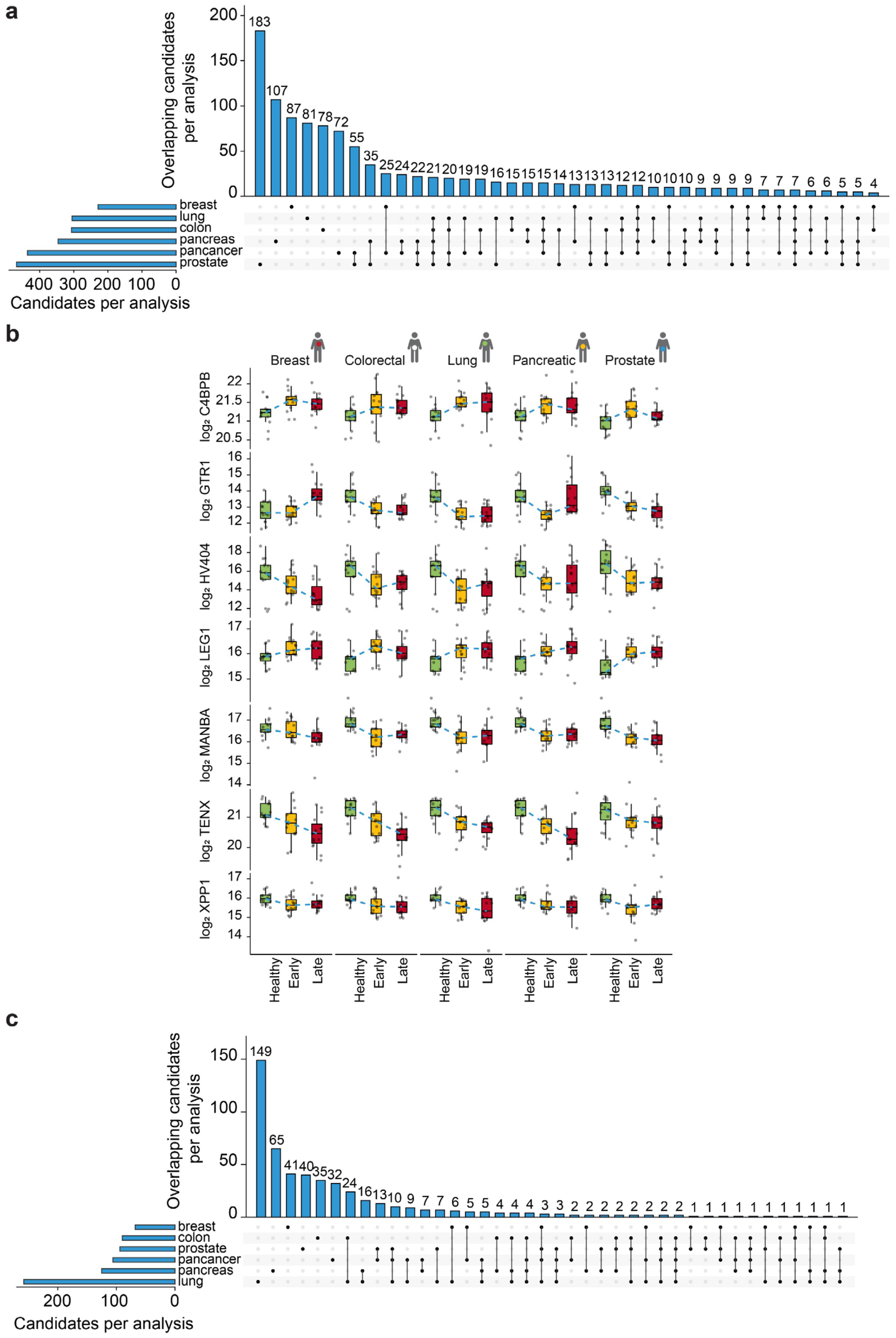

Suppl. Fig. 4: Biomarker candidates within and across the five solid cancers. (a) Set plot of proteins coming from the univariate analysis and used downstream for the different cancer models. Blue strips on the left show the number of proteins selected by pairwise comparison. Dots and lines represent subsets. The histogram represents the number of overlapping proteins in each subset. **(b)** Boxplot visualization of log-transformed C4BPB, GTR1, HV404, LEG1, MANBA, TENX and XPP1 quantities divided by stage and cancer type. The healthy samples were matched to the respective cancer type. Thick lines indicate medians, boxes indicate the 25% and 75% quantiles, whiskers extend between the median and ± (1.58 × inter-quantile range), orange lines connect the medians and each data point represents a sample (n=180). The dashed blue line connects the median values across stages. **(c)** As in panel *a* but for the final sPLDA model selections.

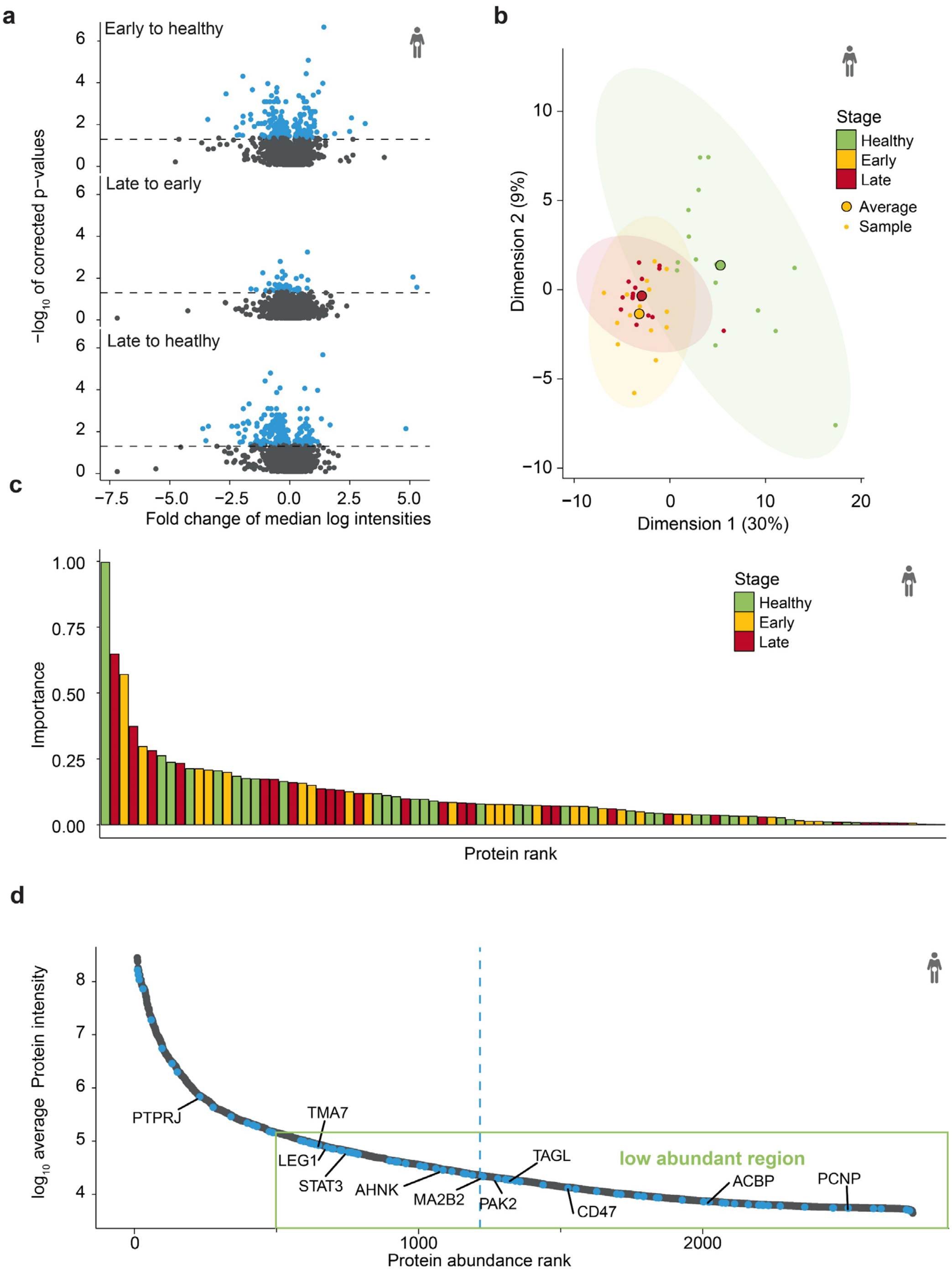

Suppl. Fig. 5: Colorectal cancer analysis. (a) Log-transformed median fold change vs. –$\log_{10}$ p-value for all proteins for the three-way comparisons (heathy, early and late stage) using univariate comparison (Pairwise Wilcoxon Rank Sum Tests) for the colorectal cancer set (n=45). The threshold for protein selection is represented as a dashed line at a p-value of 0.05. Proteins with a within-group corrected p-value below 0.05 are depicted in blue. **(b)** Representation of the first two dimensions from the PCA analysis of sPLSDA identified candidates in colorectal cancer analysis. Small points represent samples and large points the average across the stage (n=45). The first dimension separates healthy from diseased samples and explains 30% of the variance in the data. Corresponding ellipses represent sample concentration around the mean. **(c)** Representation of the sPLSDA selected biomarker candidates (90 in total) for the colorectal cancer model ordered by absolute importance and colored by the stage. **(d)** Average protein intensity plotted vs. protein abundance rank. The machine learning selected biomarkers candidates for the colorectal cancer model are colored in blue (the average is plotted as a blue line), and important contributors are highlighted. The green box depicts the proteome region that is typically below the sensitivity of native plasma profiling by mass spectrometry.

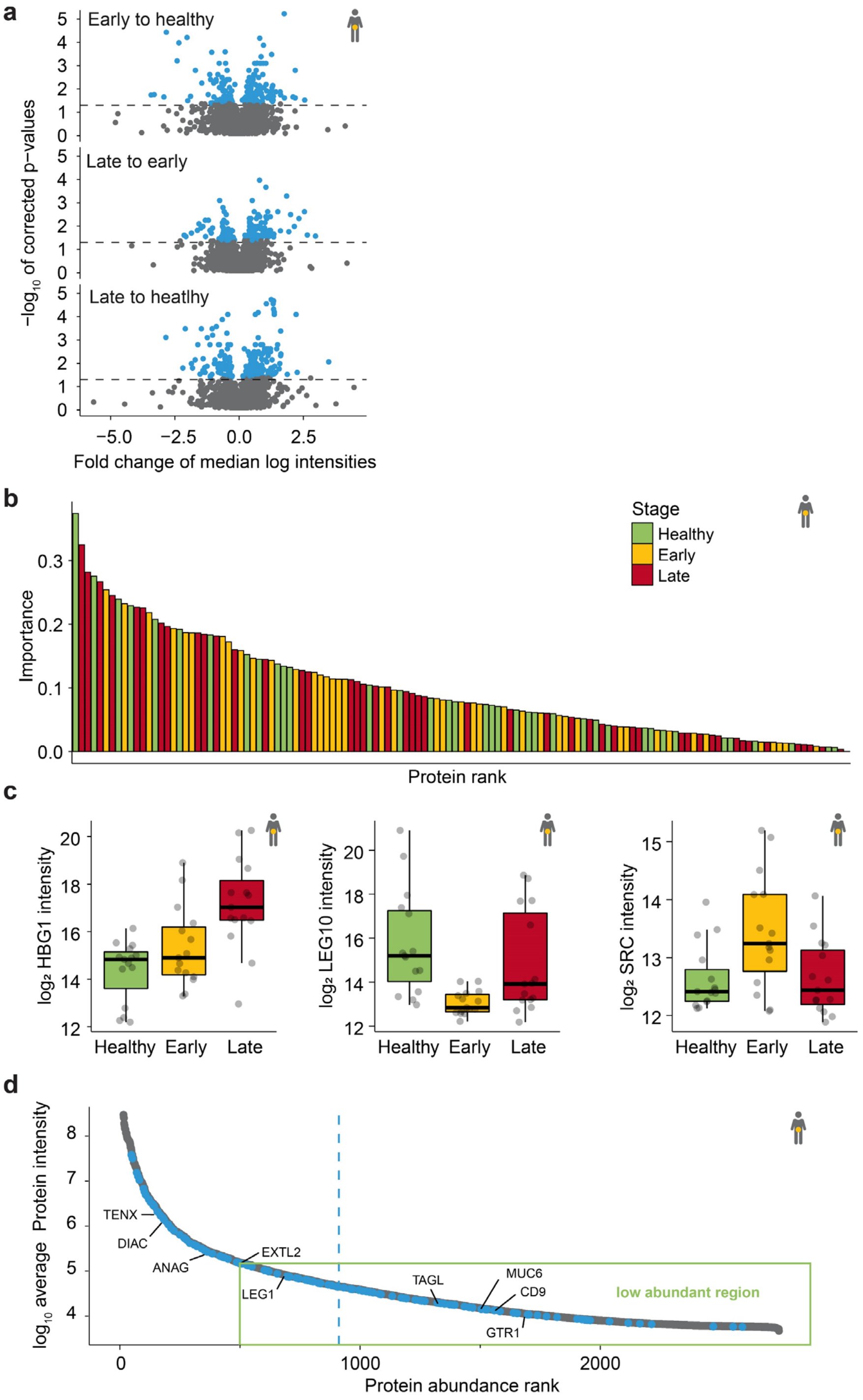

Suppl. Fig. 6: Pancreatic cancer analysis. (a) Log-transformed median fold change vs. $-\log_{10}$ p-value for all proteins for the three-way comparisons (heathy, early and late stage) using univariate comparison (Pairwise Wilcoxon Rank Sum Tests) for the pancreatic cancer set (n=45). The threshold for protein selection is represented as a dashed line at a p-value of 0.05. Proteins with a within-group corrected p-value below 0.05 are depicted in blue. **(b)** Representation of the sPLSDA selected biomarker candidates for the pancreatic cancer model (106 in total) ordered by absolute importance and colored by the stage. **(c)** Boxplot visualization of selected top candidates log-transformed HBG1, LEG10 and SRC quantities across stages for the pancreatic cancer set. Thick lines indicate medians, boxes indicate the 25% and 75% quantiles, whiskers extend between the median and ± (1.58 × inter-quantile range) and each data point represents a sample (n=45). **(d)** Average protein intensity plotted vs. protein abundance rank. The machine learning selected biomarkers candidates for the pancreatic cancer model are colored in blue (the average is plotted as a blue line) and important contributors are highlighted. The green box depicts the proteome region that is typically below the sensitivity of native plasma profiling by mass spectrometry.

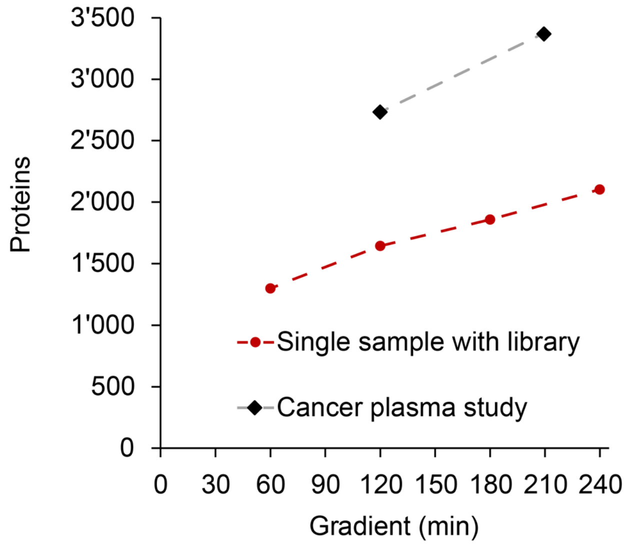

Suppl. Fig. 7: Identifications for the cancer plasma study in dependence of gradient length. The number of protein groups identified at different gradient lengths for a depleted human plasma pool using a sample specific library (red). The black diamonds shows the number of proteins identified in the presented pan-cancer study at 2h gradient length and a subset (10 healthy and 10 pancreatic cancer samples) measured at 3.5h gradient length.
